# An Efficient Framework for Estimating the Direction of Multiple Sound Sources Using Higher-Order Generalized Singular Value Decomposition

**DOI:** 10.3390/s19132977

**Published:** 2019-07-05

**Authors:** Bandhit Suksiri, Masahiro Fukumoto

**Affiliations:** 1 Department of Engineering, Graduate School of Engineering, Kochi University of Technology, Kami Campus, Kochi 782-0003, Japan; 2 School of Information, Kochi University of Technology, Kami Campus, Kochi 782-0003, Japan

**Keywords:** direction-of-arrival (DOA), higher-order generalized singular value decomposition (HOGSVD), wideband sources, sound source, array processing, subspace method, cross-correlation

## Abstract

This paper presents an efficient framework for estimating the direction-of-arrival (DOA) of wideband sound sources. The proposed framework provides an efficient way to construct a wideband cross-correlation matrix from multiple narrowband cross-correlation matrices for all frequency bins. In addition, the proposed framework is inspired by the coherent signal subspace technique with further improvement of linear transformation procedure, and the new procedure no longer requires any process of DOA preliminary estimation by exploiting unique cross-correlation matrices between the received signal and itself on distinct frequencies, along with the higher-order generalized singular value decomposition of the array of this unique matrix. Wideband DOAs are estimated by employing any subspace-based technique for estimating narrowband DOAs, but using the proposed wideband correlation instead of the narrowband correlation matrix. It implies that the proposed framework enables cutting-edge studies in the recent narrowband subspace methods to estimate DOAs of the wideband sources directly, which result in reducing computational complexity and facilitating the estimation algorithm. Practical examples are presented to showcase its applicability and effectiveness, and the results show that the performance of fusion methods perform better than others over a range of signal-to-noise ratios with just a few sensors, which make it suitable for practical use.

## 1. Introduction

The fundamental competence of sound source localization has received much attention during the past decades, and has become an important part of navigation systems [1,2]. Direction-of-arrival (DOA) estimation in particular plays a critical role in navigation systems for the exploration of sources in widespread applications, including in acoustic signal processing [3,4,5,6,7,8]. Several approaches have been proposed as a potential way to estimate DOA. For instance, the time-difference-of-arrival-based DOA estimation is one of the most frequently used approaches, which is widely known as the generalized cross-correlation with phase transform (GCC-PHAT) [9]. In addition to this approach, a low computational requirement makes it attractive for practical applications; however, the major drawback is its low robustness in noisy and multipath environments. Another relevant approach is adopted from the independent component analysis (ICA) in blind source separation [10,11]. ICA searches independent components by measuring deviations from Gaussian distributions, such as maximization of negentropy or kurtosis. DOAs are estimated easily by using the separated components for all frequency bins, but it should be noted that the estimation accuracy of such a method is highly sensitive to the non-Gaussianity measures.

In an alternative approach to estimate narrowband DOAs, the subspace method has been proposed in an effort to improve estimation performance. The most prominent methods observe the signal and noise subspace for achieving more robust results, such as multiple signal classification (MUSIC) [12], estimation of signal parameters via rotational invariance techniques (ESPRIT) [13], and propagator method [14,15], which have been used frequently for one-dimensional (1D) DOA estimation along with the uniform linear array (ULA) of sensors. In case of a two-dimensional (2D) DOA estimation, a new geometrical structure of a sensor array is required, and it was previously found that the structure of an L-shaped array is considerably effective for estimating 2D DOAs [16]. Additionally, the L-shaped array allows for simple implementation, because it consists of two ULAs connected orthogonally at one end of each ULA. For these reasons, the L-shaped array is widely applied to the 2D DOA estimation method [17,18,19,20,21,22,23,24,25,26], and its practical applications can be found in the past researches [27,28]. Although the narrowband subspace method may be unable to directly estimate wideband DOAs, one possible way to solve this problem is to employ the narrowband subspace method in each temporal frequency intensively, and then the wideband DOA results can be estimated by interpolating the narrowband DOA results all frequency bins [29,30]. It should be noted again that intensive computational costs encountered in the above solution may be limited by practical considerations.

Several approaches were proposed to solve the problem of estimating wideband DOAs, for example, the incoherent MUSIC (IMUSIC) is one of the simplest methods for estimating wideband DOA [31]. There are two steps in IMUSIC: Firstly, a noise subspace model each temporal frequency is constructed. Then, wideband DOAs are obtained by minimizing the norm of orthogonal relation between a steering vector and the noise subspace of all frequency bins. Although accuracy performance of IMUSIC was demonstrated to be an effective method for estimating DOAs of multiple wideband signals in the high signal-to-noise ratio (SNR) region, a single small distortion of the noise subspace at any frequency can affect the whole DOA results. Many attempts were made recently to overcome this problem. For instance, the test of orthogonality of frequency subspaces (TOFS) was proposed to overcome this difficulty [32], but performance degradation caused by the small distortion still remains challenging. Another relevant approach is called the test of orthogonality of projected subspaces (TOPS) [33]. TOPS estimate DOA by constructing signal subspace of one reference frequency, and then measuring orthogonality of the previous signal subspace and noise subspace for all frequency bins. The simulations showed that TOPS is able to achieve higher accuracy than IMUSIC in mid SNR range, however, the undesirable false peaks still remain. The revised and greatly improved version of TOPSs were proposed recently to reduce these false peaks [34,35]. Obviously, computational complexities increased dramatically compared to the classical TOPS.

Another notable approach of wideband DOA estimation is the coherent signal subspace method (CSS) [36,37]. CSS specifically focuses a correlation matrix of received signals of each temporal frequency into a single matrix, which is called a universal correlation, associated with one focusing frequency via linear transformation procedure. Wideband DOAs are estimated by applying a single scheme of any narrowband subspace method on the universal correlation matrix. In addition to the transformation procedure of CSS [38,39,40], a process of DOA preliminary estimation is required before the wideband DOAs can be estimated. Therefore, a common shortcoming is clearly recognized as a requirement of DOA preliminary estimation, which means that any inferior initiation can lead to biased estimates. According to the literature [31,32,33,41], CSS demonstrates deficient performance than others such as TOPS; this is because the solutions of transformation procedure in CSS are solely focused on subspace between a temporal frequency and focusing frequency; to the best knowledge of the authors, it means that a fundamental component of the transformation matrix across all frequency bins may exhibit the different core component, which is clearly apparent when a narrowband DOA result at some frequency is not close enough to the true DOA. A single component distortion can definitely affect the whole DOA results. Therefore, the solutions have to exhibit the exact component even though power present in a received signal at that frequency is very weak; in other words, the solution of transformation matrix have to be focused across all frequency bins instead of the pair of different frequencies.

Therefore, the purpose of this paper is to investigate an alternative for estimating wideband 2D DOAs in a more efficient way. We consider wideband sources as sound sources, such as human speeches and musical sounds. In order to estimate the wideband DOAs, we address the issue of transforming multiple narrowband cross-correlation matrices for all frequency bins into a wideband cross-correlation matrix. Additionally, our study is inspired by a computational model of CSS with further improvement of a linear transformation procedure [36,37,38,39,40]. Since the transformation procedures of CSS are only focused on subspace between current and reference frequency as previously mentioned, we propose a new transformation procedure which focus all frequency bins simultaneously and efficiently. The higher-order generalized singular value decomposition (HOGSVD) is firstly used to achieve this important issue [42]. By employing HOGSVD of arrays of the new unique cross-correlation matrix, where elements in the row and column positions are a sample cross-correlation matrix between received signal and itself on two distinct frequencies, the new transformation procedure no longer require any process of DOA preliminary estimation. Finally, the wideband cross-correlation matrix is constructed via the proposed transformation procedure, and the wideband DOAs can be estimated by employing any subspace-based technique for estimating narrowband DOAs, but using this wideband correlation matrix instead of the narrowband correlation matrix. Therefore, the proposed framework enables cutting-edge studies in the recent narrowband subspace methods to estimate DOA of the wideband sources directly, which result in reducing computational complexity and facilitating the estimation algorithm. Practical examples, such as 2D-MUSIC and ESPRIT with an L-shaped array, are presented to showcase its applicability and effectiveness.

The rest of this paper is organized as follows. Section 2 presents the array signal model, basic assumptions and problem formulation for transforming narrowband sample cross-correlation matrices for all frequency bins into a single matrix, which is called wideband cross-correlation matrix. Description of the new transformation procedure is introduced in Section 3.1 and its effective solution via HOGSVD in Section 3.2. Section 3.3 provide a description of the proposed framework for estimating wideband DOAs by combining the proposed transformation procedure along with a scheme of estimating DOAs in a recent narrowband subspace method, and its practical examples are presented in Section 3.3.1 and Section 3.3.2. The simulation and experimental results are compared with the several existing methods in Section 4 and Section 5. Finally, Section 6 concludes this paper.

## 2. Preliminaries

### 2.1. Data Model

The proposed method presented in this paper considers far-field sound sources. Received signals are a composition of the multiple sources, each one consisting of an angle in a spherical coordinate system. The received signals are transformed into a time-frequency representation via the short-time Fourier transform (STFT), and are given by

(1) $$r\left(t,f\right)=A\left(\phi ,\theta ,f\right)s\left(t,f\right)+w\left(t,f\right),$$

where $r\left(t,f\right)\in C^{M}$ is the summation of a received signal, $s\left(t,f\right)\in C^{K}$ is a source signal, $w\left(t,f\right)\in C^{M}$ is an additive noise, the constant *M* is the number of microphone elements, and *K* is the number of incident sources. The matrix $A\left(\phi ,\theta ,f\right)\in C^{M\times K}$ stands for the array manifold where ϕ and θ are phase angle components of the source on *x* and *z* axes in the spherical coordinate system. Note that the elements in $A\left(\phi ,\theta ,f\right)$ depend on an array geometry.

**Figure 1:**
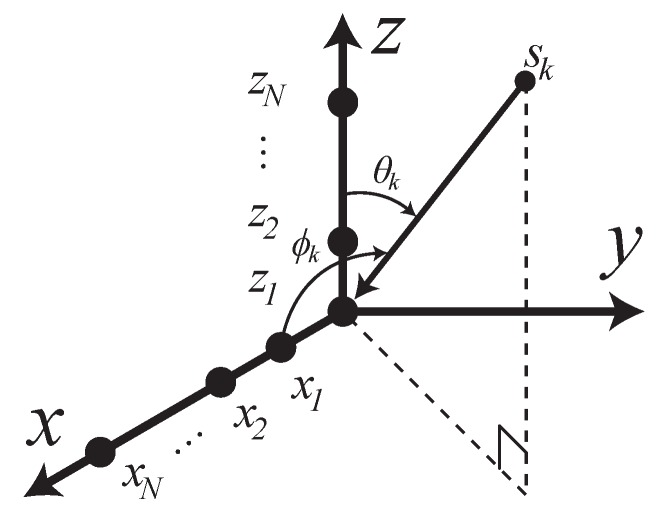
L-shaped microphone array configuration for 2D DOA estimation.

Consider the L-shaped array structure consisting of two ULAs as illustrated in Figure 1, the received signals are simplified as

(2) $$\left[\begin{array}{c} x\left(t,f\right)\\ z\left(t,f\right) \end{array}\right]=\left[\begin{array}{c} A_{x}\left(\phi ,f\right)\\ A_{z}\left(\theta ,f\right) \end{array}\right]s\left(t,f\right)+\left[\begin{array}{c} w_{x}\left(t,f\right)\\ w_{z}\left(t,f\right) \end{array}\right],$$

where

(3) $$\begin{array}{cc} A_{x}\left(\phi ,f\right) & =\left[\begin{array}{cccc} a_{x}\left(\phi_{1},f\right) & a_{x}\left(\phi_{2},f\right) & \cdots & a_{x}\left(\phi_{K},f\right) \end{array}\right],\\ A_{z}\left(\theta ,f\right) & =\left[\begin{array}{cccc} a_{z}\left(\theta_{1},f\right) & a_{z}\left(\theta_{2},f\right) & \cdots & a_{z}\left(\theta_{K},f\right) \end{array}\right],\\ a_{x}\left(\phi_{k},f\right) & ={\left[\begin{array}{cccc} e^{\alpha_{x}\left(\phi_{k},f\right)j} & e^{2\alpha_{x}\left(\phi_{k},f\right)j} & \cdots & e^{N\alpha_{x}\left(\phi_{k},f\right)j} \end{array}\right]}^{T},\\ a_{z}\left(\theta_{k},f\right) & ={\left[\begin{array}{cccc} 1 & e^{\alpha_{z}\left(\theta_{k},f\right)j} & \cdots & e^{\left(N-1\right)\alpha_{z}\left(\theta_{k},f\right)j} \end{array}\right]}^{T},\\ \alpha_{x}\left(\phi_{k},f\right) & =\left(\frac{f}{f_{o}}\right)\;\cdot \;\left(\frac{2\pi d\cos\phi_{k}}{\lambda }\right),\\ \alpha_{z}\left(\theta_{k},f\right) & =\left(\frac{f}{f_{o}}\right)\;\cdot \;\left(\frac{2\pi d\cos\theta_{k}}{\lambda }\right). \end{array}$$

From the above definitions, $x\left(t,f\right),w_{x}\left(t,f\right)\in C^{N},A_{x}\left(\phi ,f\right)\in C^{N\times K}$ and a subscript *x* are belonged to *x* subarray, and likewise, $z\left(t,f\right),w_{z}\left(t,f\right)\in C^{N},A_{z}\left(\theta ,f\right)\in C^{N\times K}$ and a subscript *z* are belonged to *z* subarray where *N* is the number of microphone elements each subarray with M=2N. The variable *t* is time, *f* is a source frequency, *d* is the spacing of microphone elements, λ is a wavelength with respect to $\lambda =\frac{c}{f_{o}}$ where *c* is the speed of sound in current medium, and $f_{o}$ is a reference frequency.

### 2.2. Basic Assumptions

Based on the recent reviews, the following assumptions are required on the proposed framework:

Assumption 1: The number of sources is known or predicted in advance [43,44].

Assumption 2: The spacing between adjacent elements of each subarray and spacing between $x_{1}$ and $z_{1}$ should be set to $d=\frac{\lambda }{2}$ for avoiding the angle ambiguity in array structure radiation [1,2,16].

Assumption 3: The source $s\left(t,f\right)$ is assumed to be Gaussian complex random variable as suggested by the literature [12,16,31]. However, we consider wideband sources as sound sources such as human speech; therefore, $s\left(t,f\right)$ can also be Super-Gaussian complex random variable, and it is not stationary signals for the most general case when giving an appropriate period of time.

Assumption 4: According to acoustic theory of speech, frequency dependence of the sound source, especially a human speech, is existed [45]; it means that a cross-covariance between the source and itself with distinct frequencies is not zero; $\mathrm{cov}\left(s_{k}\left(t,f\right),s_{k}\left(t,f^{\prime }\right)\right)=c_{s_{k}\left\{f,f^{\prime }\right\}},$ where $c_{s_{k}\left\{f,f^{\prime }\right\}}\in C$. Next, suppose that $s\left(t,f\right)$ are uncorrelated, which implies that $s_{k}\left(t,f\right)$ and $s_{k^{\prime }}\left(t,f^{\prime }\right)$ are statistically independent of each other when $k\neq k^{\prime }$; $\mathrm{cov}\left(s_{k}\left(t,f\right),s_{k^{\prime }}\left(t,f^{\prime }\right)\right)=0$. When $k=k^{\prime }$, the sources can take to be partially dependent by the following literature [45]; therefore, a sample cross-covariance matrix of the incident sources over two different frequencies is given by

(4) $$\begin{array}{cc} S_{\left\{f,f^{\prime }\right\}} & =E\left\{s\left(t,f\right)s^{H}\left(t,f^{\prime }\right)\right\}\\ & =\mathrm{diag}\left(c_{s_{1}\left\{f,f^{\prime }\right\}},c_{s_{2}\left\{f,f^{\prime }\right\}},\ldots ,c_{s_{k}\left\{f,f^{\prime }\right\}}\right). \end{array}$$

Remark that $c_{s_{k}\left\{f,f\right\}}$ is equal to $\sigma_{s_{k}\left\{f\right\}}^{2}$, and $\sigma_{s_{k}\left\{f\right\}}^{2}\in R_{\geq 0}$ is a variance at frequency *f* of the source.

Assumption 5: An additive white Gaussian noise is considered in this paper, which is modeled as Gaussian random variable as well as the past studies. A noise cross-covariance matrix over two different frequencies is given by

(5) $$\begin{array}{cc} W_{\left\{f,f^{\prime }\right\}} & =E\left\{w\left(t,f\right)w^{H}\left(t,f^{\prime }\right)\right\}\\ & =c_{w\left\{f,f^{\prime }\right\}}I_{M}, \end{array}$$

where $c_{w\left\{f,f^{\prime }\right\}}\in C$, and $I_{i}$ is a *i*-by-*i* identity matrix. Note again that $c_{w\left\{f,f\right\}}=\sigma_{w\left\{f\right\}}^{2}$ where $\sigma_{w\left\{f\right\}}^{2}\in R_{\geq 0}$ is a variance of the noise at frequency *f*. In case of the L-shaped array structure in Equation (2), we have

(6) $$\left[\begin{array}{cc} W_{xx\left\{f,f^{\prime }\right\}} & W_{xz\left\{f,f^{\prime }\right\}}\\ W_{zx\left\{f,f^{\prime }\right\}} & W_{zz\left\{f,f^{\prime }\right\}} \end{array}\right]=\left[\begin{array}{cc} c_{w\left\{f,f^{\prime }\right\}}I_{N} & O_{N\times N}\\ O_{N\times N} & c_{w\left\{f,f^{\prime }\right\}}I_{N} \end{array}\right],$$

where $O_{i\times j}$ is a *i*-by-*j* null matrix.

### 2.3. Transformation Problem

Under the data model and assumptions in Section 2.1 and Section 2.2, a cross-correlation matrix of the received signals is defined as

(7) $$\begin{array}{cc} R_{\left\{f,f^{\prime }\right\}} & =E\left\{r\left(t,f\right)r^{H}\left(t,f^{\prime }\right)\right\}\\ & =A\left(\phi ,\theta ,f\right)S_{\left\{f,f^{\prime }\right\}}A^{H}\left(\phi ,\theta ,f^{\prime }\right)+W_{\left\{f,f^{\prime }\right\}}, \end{array}$$

where $R_{\left\{f,f^{\prime }\right\}}\in C^{M\times M}$. In order to transform $R_{\left\{f,f\right\}}$ over the available frequency range into a single smoothed matrix, which is named as a wideband cross-correlation, a transformation procedure is required as mentioned previously [36], which is expressed as

(8) $$\begin{array}{cc} R & =\frac{1}{P}\sum_{i=1}^{P}T_{\left\{f_{i}\right\}}R_{\left\{f_{i},f_{i}\right\}}T_{\left\{f_{i}\right\}}^{H}\\ & =A\left(\phi ,\theta ,f_{o}\right)\left(\frac{1}{P}\sum_{i=1}^{P}S_{\left\{f_{i},f_{i}\right\}}\right)A^{H}\left(\phi ,\theta ,f_{o}\right)+\frac{1}{P}\sum_{i=1}^{P}T_{\left\{f_{i}\right\}}W_{\left\{f_{i},f_{i}\right\}}T_{\left\{f_{i}\right\}}^{H}, \end{array}$$

where

(9) $$A\left(\phi ,\theta ,f_{o}\right)=T_{\left\{f_{i}\right\}}A\left(\phi ,\theta ,f_{i}\right),$$

$R\in C^{M\times M}$ is the wideband cross-correlation matrix, and *P* is the number of STFT frequency bins. $T_{\left\{f_{i}\right\}}\in C^{M\times M}$ is a transformation matrix, which was originally designed by using the ordinary beamforming technique [36], or by minimizing the Frobenius norm of array manifold matrices [37]. The objective of $T_{\left\{f\right\}}$ is to transform any given *f* of the array manifold $A\left(\phi ,\theta ,f\right)$ into $A\left(\phi ,\theta ,f_{o}\right)$. All previous solutions of $T_{\left\{f\right\}}$ are solely based on subspace between pair of distinct frequencies $\left\{f,f_{o}\right\}$, as emphasized in the introduction [36,37,38,39,40]. When power of the source at some frequency is weak or less than noise power, the matrix $T_{\left\{f\right\}}$ may not share any common angle of ϕ,θ because its non-zero eigenvalues are not full rank, which is resulted in a performance degradation for estimating both $T_{\left\{f\right\}}$ and wideband DOAs. If the transformation matrix can be focused by all frequency bins instead of the pair of frequencies, a good estimate of DOAs in Equation (8) might be expected. Based on this hypothesis, a new concept and scheme are presented in next section.

## 3. Proposed Method

This section introduces a new procedure for estimating a transformation matrix, its alternative solution by using the higher-order generalized singular value decomposition (HOGSVD), and practical examples of wideband DOA estimation scheme.

### 3.1. Problem for Estimating the Transformation Matrix and Its Solution

We start by introducing the following lemma that will be useful for obtaining a solution of transformation matrix.

**Lemma** **1.** *Given a set of two distinct frequencies by $\left\{f,f_{o}\right\}$ into Equation (7), and given a transformation matrix $T_{\left\{f\right\}}$ which satisfy the property in Equation (9), assume that K<M, the cross-correlation $R_{\left\{f,f_{o}\right\}}$ can be factorized into the singular value decomposition (SVD) form;*

(10) $$R_{\left\{f,f_{o}\right\}}=U_{{\left\{f,f_{o}\right\}}_{s}}\Sigma_{{\left\{f,f_{o}\right\}}_{s}}V_{{\left\{f,f_{o}\right\}}_{s}}^{H}+U_{{\left\{f,f_{o}\right\}}_{n}}\Sigma_{{\left\{f,f_{o}\right\}}_{n}}V_{{\left\{f,f_{o}\right\}}_{n}}^{H},$$

*where $U_{{\left\{f,f_{o}\right\}}_{s}},V_{{\left\{f,f_{o}\right\}}_{s}}\in C^{M\times K}$, $\Sigma_{{\left\{f,f_{o}\right\}}_{s}}\in R^{K\times K}$ are the matrix of left and right singular vectors and diagonal matrix of singular values in signal subspace, and likewise, $U_{{\left\{f,f_{o}\right\}}_{n}},V_{{\left\{f,f_{o}\right\}}_{n}}\in C^{M\times M-K}$, $\Sigma_{{\left\{f,f_{o}\right\}}_{n}}\in C^{M-K\times M-K}$ are with noise subspace. If the K largest singular values of $T_{\left\{f\right\}}R_{\left\{f,f_{o}\right\}}$ and $R_{\left\{f,f_{o}\right\}}$ are equal, then $T_{\left\{f\right\}}U_{{\left\{f,f_{o}\right\}}_{s}}$ is a matrix with orthonormal columns.*

**Proof.**  Since the transformation procedure of $R_{\left\{f,f_{o}\right\}}$ is expressed by $T_{\left\{f\right\}}R_{\left\{f,f_{o}\right\}}$ and the array manifold $A\left(\phi ,\theta ,f\right)$ and $A\left(\phi ,\theta ,f_{o}\right)$ are full rank matrices [36], Lemma 1 is valid if and only if the *K* largest singular values of $T_{\left\{f\right\}}R_{\left\{f,f_{o}\right\}}$ and $R_{\left\{f,f_{o}\right\}}$ are equal; therefore, $U_{{\left\{f,f_{o}\right\}}_{s}}T_{\left\{f\right\}}^{H}T_{\left\{f\right\}}U_{{\left\{f,f_{o}\right\}}_{s}}=I_{K}$. Considering the M−K smallest singular values of $R_{\left\{f,f_{o}\right\}}$ are close to zeros by assuming a noise-free signal and using solely the signal subspace $U_{{\left\{f,f_{o}\right\}}_{s}}\Sigma_{{\left\{f,f_{o}\right\}}_{s}}V_{{\left\{f,f_{o}\right\}}_{s}}^{H}$, we have

(11) $$\begin{array}{c} {\left(T_{\left\{f\right\}}\left(R_{\left\{f,f_{o}\right\}}-W_{\left\{f,f_{o}\right\}}\right)\right)}^{H}\left(T_{\left\{f\right\}}\left(R_{\left\{f,f_{o}\right\}}-W_{\left\{f,f_{o}\right\}}\right)\right)\\ =V_{{\left\{f,f_{o}\right\}}_{s}}\Sigma_{{\left\{f,f_{o}\right\}}_{s}}\Sigma_{{\left\{f,f_{o}\right\}}_{s}}V_{{\left\{f,f_{o}\right\}}_{s}}^{H}. \end{array}$$

Performing the Eigenvalue decomposition (EVD) to Equation (11), square roots of the non-zero eigenvalues of above matrix is equal to $\Sigma_{{\left\{f,f_{o}\right\}}_{s}}$ [46,47]. This completes the proof of the lemma. □

Lemma 1 shows that $R_{\left\{f,f_{o}\right\}}$ and $T_{\left\{f\right\}}R_{\left\{f,f_{o}\right\}}$ share the common components on the singular values and right singular vectors, whereas the both left singular vectors may be different. Since $A\left(\phi ,\theta ,f\right)$ and $A\left(\phi ,\theta ,f_{o}\right)$ are full rank, its remaining components are given by [48]:

(12) $$\begin{array}{cc} A\left(\phi ,\theta ,f\right) & =U_{{\left\{f,f_{o}\right\}}_{s}}F_{\left\{f,f_{o}\right\}},\\ T_{\left\{f\right\}}A\left(\phi ,\theta ,f\right) & =\left(T_{\left\{f\right\}}U_{{\left\{f,f_{o}\right\}}_{s}}\right)F_{\left\{f,f_{o}\right\}},\\ A\left(\phi ,\theta ,f_{o}\right) & =V_{{\left\{f,f_{o}\right\}}_{s}}G_{\left\{f,f_{o}\right\}}, \end{array}$$

where

(13) $$\Sigma_{{\left\{f,f_{o}\right\}}_{s}}=F_{\left\{f,f_{o}\right\}}S_{\left\{f,f_{o}\right\}}G_{\left\{f,f_{o}\right\}}^{H},$$

$F_{\left\{f,f_{o}\right\}},G_{\left\{f,f_{o}\right\}}\in C^{K\times K}$ are full rank matrices and have invertibility. From Equations (9) and (12), we have

(14) $$T_{\left\{f\right\}}U_{{\left\{f,f_{o}\right\}}_{s}}=V_{{\left\{f,f_{o}\right\}}_{s}}G_{\left\{f,f_{o}\right\}}F_{\left\{f,f_{o}\right\}}^{-1},$$

which mean that the right singular vectors of $R_{\left\{f,f_{o}\right\}}$ and the left singular vectors of $T_{\left\{f\right\}}R_{\left\{f,f_{o}\right\}}$ share the common subspace when $G_{\left\{f,f_{o}\right\}}F_{\left\{f,f_{o}\right\}}^{-1}$ has unitary property.

Since the left singular vectors of $T_{\left\{f\right\}}R_{\left\{f,f_{o}\right\}}$ exist, we continue to introduce a new transformation procedure. The matrix $T_{\left\{f\right\}}$ can be found as a solution to

(15) $$\begin{array}{cc} \underset{T_{\left\{f\right\}}}{\mathrm{minimize}} & {∥R_{\left\{f_{o},f_{o}\right\}}-T_{\left\{f\right\}}R_{\left\{f,f_{o}\right\}}∥}_{F}^{2}\\ \mathrm{subject}\;\mathrm{to} & \begin{array}{c} \sum_{k=1}^{K}\sigma_{k}^{2}\left(T_{\left\{f\right\}}R_{\left\{f,f_{o}\right\}}\right)=\sum_{k=1}^{K}\sigma_{k}^{2}\left(R_{\left\{f,f_{o}\right\}}\right), \end{array} \end{array}$$

where ${∥\;\cdot \;∥}_{F}$ is the Frobenius norm, and $\sum_{k=1}^{K}\sigma_{k}^{2}\left(A\right)$ is the sum-of-squares *K* largest singular values of A. If the constraint in Equation (15) is not imposed, then one of the possible choices is obtained by the least squares problem [49,50]; the solution is derived by observing the point where the derivative of cost function with respect to $T_{\left\{f\right\}}$ is zero, then we can have $T_{{\left\{f\right\}}_{\mathrm{LS}}}=R_{\left\{f_{o},f_{o}\right\}}R_{\left\{f,f_{o}\right\}}^{H}{\left(R_{\left\{f,f_{o}\right\}}R_{\left\{f,f_{o}\right\}}^{H}\right)}^{-1}$, and $\Sigma_{{\left\{f_{o},f_{o}\right\}}_{s}}\Sigma_{{\left\{f,f_{o}\right\}}_{s}}^{-1}=I_{K}$, which is difficult in practice. To solve the problem much more practically, an alternative solution is introduced, which is based on the constraint in Equation (15) and Lemma 1:

**Theorem** **1.** *Let $U_{\psi \left\{f\right\}},V_{\psi \left\{f\right\}}\in C^{M\times K}$ are the matrices in signal subspace containing the left and right singular vectors of $R_{\left\{f,f_{o}\right\}}R_{\left\{f_{o},f_{o}\right\}}^{H}$. Imposing the constraint in Equation (15) and Lemma 1, along with the modification of orthogonal Procrustes problem (MOP), an alternative solution to Equation (15) is given by*

(16) $$T_{{\left\{f\right\}}_{\mathrm{MOP}}}=V_{\psi \left\{f\right\}}U_{\psi \left\{f\right\}}^{†},$$

*where † stands for the pseudo-inverse of a matrix. Defining the square matrix $\Omega_{\left\{f\right\}}\in C^{K\times K}$ as the matrix containing error corrections, the error of transformation remains consistent with the following equation;*

(17) $$\varepsilon_{{\left\{f\right\}}_{\mathrm{MOP}}}=\left|2\;\cdot \;ℜ\left(tr\left(\Sigma_{\psi \left\{f\right\}}\left(\Omega_{\left\{f\right\}}-I_{K}\right)\right)\right)+tr\left(\Sigma_{{\left\{f,f_{o}\right\}}_{n}}^{2}{\left(U_{\psi \left\{f\right\}}^{†}U_{\varepsilon \left\{f\right\}}\right)}^{H}U_{\psi \left\{f\right\}}^{†}U_{\varepsilon \left\{f\right\}}\right)\right|$$

*where $\Sigma_{\psi \left\{f\right\}}\in R^{K\times K}$ and $U_{\varepsilon \left\{f\right\}}\in C^{M\times M-K}$ are the diagonal matrix of the K largest singular values, and the noise subspace left singular vectors of $R_{\left\{f,f_{o}\right\}}R_{\left\{f_{o},f_{o}\right\}}$, respectively.*

**Proof.**  See Appendix A. □

Theorem 1 provides an efficient way to construct $T_{\left\{f\right\}}$ without any process of DOA preliminary estimation, but the solution are still solely based on subspace between pair of distinct frequencies. In order to observe the solution across all frequency bins, we will present an alternative for constructing $T_{\left\{f\right\}}$ by using HOGSVD along with Theorem 1, which the next section will address further.

### 3.2. Estimation of the Transformation Matrices by HOGSVD

Suppose we have a set of *P* complex matrices $E_{\left\{f_{i}\right\}}\in C^{M\times M}$ and all of them have a full rank;

(18) $$\begin{array}{cc} E_{\left\{f_{1}\right\}} & =R_{\left\{f_{1},f_{o}\right\}}R_{\left\{f_{o},f_{o}\right\}}^{H},\\ E_{\left\{f_{2}\right\}} & =R_{\left\{f_{2},f_{o}\right\}}R_{\left\{f_{o},f_{o}\right\}}^{H},\\ \;\vdots \\ E_{\left\{f_{P}\right\}} & =R_{\left\{f_{P},f_{o}\right\}}R_{\left\{f_{o},f_{o}\right\}}^{H}, \end{array}$$

where $\left\{f_{1},f_{2},\cdots ,f_{P}\right\}$ is a set of frequency intervals, and the cross-correlation matrices $R_{\left\{f_{i},f_{o}\right\}}$ and $R_{\left\{f_{o},f_{o}\right\}}$ are obtained form Equation (7). The definition of HOGSVD of these *P* matrices are given by the generalized singular value decomposition (GSVD) of P≥2 datasets and its right singular vectors are identical in all decomposition [42], as follows:

(19) $$\left[\begin{array}{c} E_{\left\{f_{1}\right\}}\\ E_{\left\{f_{2}\right\}}\\ \vdots \\ E_{\left\{f_{P}\right\}} \end{array}\right]=\left[\begin{array}{c} U_{e{\left\{f_{1}\right\}}_{s}}\Sigma_{e{\left\{f_{1}\right\}}_{s}}\\ U_{e{\left\{f_{2}\right\}}_{s}}\Sigma_{e{\left\{f_{2}\right\}}_{s}}\\ \vdots \\ U_{e{\left\{f_{P}\right\}}_{s}}\Sigma_{e{\left\{f_{P}\right\}}_{s}} \end{array}\right]V_{e_{s}}^{H}+\left[\begin{array}{c} U_{e{\left\{f_{1}\right\}}_{n}}\Sigma_{e{\left\{f_{1}\right\}}_{n}}\\ U_{e{\left\{f_{2}\right\}}_{n}}\Sigma_{e{\left\{f_{2}\right\}}_{n}}\\ \vdots \\ U_{e{\left\{f_{P}\right\}}_{n}}\Sigma_{e{\left\{f_{P}\right\}}_{n}} \end{array}\right]V_{e_{n}}^{H},$$

where $U_{e{\left\{f_{i}\right\}}_{s}}\in C^{M\times K}$, $U_{e{\left\{f_{i}\right\}}_{n}}\in C^{M\times M-K}$ are the matrix of left singular vectors, $V_{e_{s}}\in C^{M\times K}$, $V_{e_{n}}\in C^{M\times M-K}$ are the matrix of right singular vectors, and $\Sigma_{e{\left\{f_{i}\right\}}_{s}}\in R^{K\times K}$, $\Sigma_{e{\left\{f_{i}\right\}}_{n}}\in R^{M-K\times M-K}$ are the diagonal matrix of singular values. Note that subscripts *s* and *n* denote subspace of signal and noise, respectively. Unlike the left singular vectors $U_{{\left\{f,f_{o}\right\}}_{s}}$ and $U_{{\left\{f,f_{o}\right\}}_{n}}$ that have orthonormal columns by performing SVD, $U_{e{\left\{f_{i}\right\}}_{s}}$ and $U_{e{\left\{f_{i}\right\}}_{n}}$ now have unit 2-norm columns instead.

To show that $V_{e_{s}}$ is equal to $V_{\psi {\left\{f\right\}}_{s}}$ for all frequency bins, let us start from brief description of HOGSVD benchmark. The matrix $V_{e_{s}}$ is obtained by performing EVD on the following matrix;

(20) $$\begin{array}{cc} S & =\frac{1}{P\left(P-1\right)}\sum_{i=1}^{P}\sum_{j=i+1}^{P}\left(\left(E_{\left\{f_{i}\right\}}^{H}E_{\left\{f_{i}\right\}}\right){\left(E_{\left\{f_{j}\right\}}^{H}E_{\left\{f_{j}\right\}}\right)}^{-1}+\left(E_{\left\{f_{j}\right\}}^{H}E_{\left\{f_{j}\right\}}\right){\left(E_{\left\{f_{i}\right\}}^{H}E_{\left\{f_{i}\right\}}\right)}^{-1}\right). \end{array}$$

Let us redefine

(21) $$\begin{array}{cc} E_{\left\{f_{i}\right\}} & =U_{o\left\{f_{i}\right\}}\Sigma_{o\left\{f_{i}\right\}}V_{o\left\{f_{i}\right\}}^{H}, \end{array}$$

where

(22) $$\begin{array}{cc} U_{o\left\{f_{i}\right\}} & =\left[\begin{array}{cc} U_{\psi \left\{f_{i}\right\}} & U_{\varepsilon \left\{f_{i}\right\}} \end{array}\right],\\ V_{o\left\{f_{i}\right\}} & =\left[\begin{array}{cc} V_{\psi \left\{f_{i}\right\}} & V_{\varepsilon \left\{f_{i}\right\}} \end{array}\right],\\ \Sigma_{o\left\{f_{i}\right\}} & =\left[\begin{array}{cc} \Sigma_{\psi \left\{f_{i}\right\}} & O_{K\times M-K}\\ O_{M-K\times K} & \Sigma_{\varepsilon \left\{f_{i}\right\}} \end{array}\right], \end{array}$$

$\Sigma_{\varepsilon \left\{f_{i}\right\}}\in R^{M-K\times M-K}$ is the matrix of the M−K smallest singular values of $R_{\left\{f_{i},f_{o}\right\}}R_{\left\{f_{o},f_{o}\right\}}^{H}$, and $V_{\psi \left\{f_{i}\right\}}=Q_{{\left\{f_{o}\right\}}_{s}}$, $V_{\varepsilon \left\{f_{i}\right\}}=Q_{{\left\{f_{o}\right\}}_{n}}$ by employing Theorem 1 (For details, see Appendix A). Substituting Equations (21) and (22) into Equation (20), we have

(23) $$\begin{array}{cc} \left(E_{\left\{f_{i}\right\}}^{H}E_{\left\{f_{i}\right\}}\right){\left(E_{\left\{f_{j}\right\}}^{H}E_{\left\{f_{j}\right\}}\right)}^{-1} & =V_{o\left\{f_{i}\right\}}\Sigma_{o\left\{f_{i}\right\}}\Sigma_{o\left\{f_{i}\right\}}\Sigma_{o\left\{f_{j}\right\}}^{-1}\Sigma_{o\left\{f_{j}\right\}}^{-1}V_{o\left\{f_{j}\right\}}^{-1},\\ \left(E_{\left\{f_{j}\right\}}^{H}E_{\left\{f_{j}\right\}}\right){\left(E_{\left\{f_{i}\right\}}^{H}E_{\left\{f_{i}\right\}}\right)}^{-1} & =V_{o\left\{f_{j}\right\}}\Sigma_{o\left\{f_{j}\right\}}\Sigma_{o\left\{f_{j}\right\}}\Sigma_{o\left\{f_{i}\right\}}^{-1}\Sigma_{o\left\{f_{i}\right\}}^{-1}V_{o\left\{f_{i}\right\}}^{-1}. \end{array}$$

Since $V_{\psi \left\{f_{i}\right\}}=Q_{{\left\{f_{o}\right\}}_{s}}=V_{\psi \left\{f_{j}\right\}}$, $V_{\varepsilon \left\{f_{i}\right\}}=Q_{{\left\{f_{o}\right\}}_{n}}=V_{\varepsilon \left\{f_{i}\right\}}$ for all frequency bins, therefore

(24) $$\begin{array}{cc} S & =V_{e}\left(\frac{1}{P\left(P-1\right)}\sum_{i=1}^{P}\sum_{j=i+1}^{P}\left(\Sigma_{o\left\{f_{i}\right\}}\Sigma_{o\left\{f_{i}\right\}}\Sigma_{o\left\{f_{j}\right\}}^{-1}\Sigma_{o\left\{f_{j}\right\}}^{-1}+\Sigma_{o\left\{f_{j}\right\}}\Sigma_{o\left\{f_{j}\right\}}\Sigma_{o\left\{f_{i}\right\}}^{-1}\Sigma_{o\left\{f_{i}\right\}}^{-1}\right)\right)V_{e}^{-1}. \end{array}$$

where

(25) $$V_{e}=\left[\begin{array}{cc} Q_{{\left\{f_{o}\right\}}_{s}} & Q_{{\left\{f_{o}\right\}}_{n}} \end{array}\right].$$

Preforming EVD in Equation (24), we can obtain $V_{e_{s}}$, which reveal that $V_{e_{s}}$ is equal to $V_{\psi \left\{f\right\}}$ for all frequency bins. In addition, it can be seen that the matrix $V_{e_{s}}$ or $V_{\psi \left\{f\right\}}$ is estimated by focusing all frequency bins simultaneously; when power of the source at some frequency is weak or less than noise power, the matrices $V_{\psi \left\{f\right\}}$ still share common angle of ϕ,θ across all frequency bands effectively and identically.

After obtaining the right singulars vectors of $E_{\left\{f_{i}\right\}}$, we then moved forward to find its left singulars vectors. We start by considering the following equations based in Equations (19) and (25);

(26) $$\begin{array}{cc} \left[\begin{array}{c} E_{\left\{f_{1}\right\}}\\ E_{\left\{f_{2}\right\}}\\ \vdots \\ E_{\left\{f_{P}\right\}} \end{array}\right]V_{e} & =\left[\begin{array}{c} U_{e{\left\{f_{1}\right\}}_{s}}\Sigma_{e{\left\{f_{1}\right\}}_{s}}\\ U_{e{\left\{f_{2}\right\}}_{s}}\Sigma_{e{\left\{f_{2}\right\}}_{s}}\\ \vdots \\ U_{e{\left\{f_{P}\right\}}_{s}}\Sigma_{e{\left\{f_{P}\right\}}_{s}} \end{array}\right]\left[\begin{array}{cc} I_{K} & O_{K\times M-K} \end{array}\right]+\left[\begin{array}{c} U_{e{\left\{f_{1}\right\}}_{n}}\Sigma_{e{\left\{f_{1}\right\}}_{n}}\\ U_{e{\left\{f_{2}\right\}}_{n}}\Sigma_{e{\left\{f_{2}\right\}}_{n}}\\ \vdots \\ U_{e{\left\{f_{P}\right\}}_{n}}\Sigma_{e{\left\{f_{P}\right\}}_{n}} \end{array}\right]\left[\begin{array}{cc} O_{M-K\times K} & I_{M-K} \end{array}\right]. \end{array}$$

We remark again that $U_{e{\left\{f_{i}\right\}}_{s}}$, $U_{e{\left\{f_{i}\right\}}_{n}}$ have unit 2-norm columns instead of orthonormal columns [42];

(27) $$\left[\begin{array}{c} U_{e{\left\{f_{i}\right\}}_{s}}^{H}\\ U_{e{\left\{f_{i}\right\}}_{n}}^{H} \end{array}\right]\left[\begin{array}{cc} U_{e{\left\{f_{i}\right\}}_{s}} & U_{e{\left\{f_{i}\right\}}_{n}} \end{array}\right]=\left[\begin{array}{cccc} 1 & \xi_{12} & \cdots & \xi_{1M}\\ \xi_{21} & 1 & \cdots & \xi_{2M}\\ \vdots & \vdots & \ddots & \vdots \\ \xi_{M1} & \xi_{M2} & \cdots & 1 \end{array}\right],$$

where $\xi_{jk}\in C,\;\forall j\in M,\forall k\in M:j\neq k$. Then, the singular values are obtained as follows:

(28) $$\begin{array}{cc} \Sigma_{e{\left\{f_{i}\right\}}_{s}} & =\mathrm{diag}\left({∥e_{1}∥}_{2},{∥e_{2}∥}_{2},\cdots ,{∥e_{K}∥}_{2}\right),\\ \Sigma_{e{\left\{f_{i}\right\}}_{n}} & =\mathrm{diag}\left({∥e_{K+1}∥}_{2},{∥e_{K+2}∥}_{2},\cdots ,{∥e_{M}∥}_{2}\right), \end{array}$$

where ${∥\;\cdot \;∥}_{2}$ is the Euclidean norm, and $e_{j}\in C^{M}$ is a $j^{\mathrm{th}}$ column of $E_{\left\{f_{i}\right\}}V_{e}$. Finally, the matrices $U_{e{\left\{f_{i}\right\}}_{s}}$, $U_{e{\left\{f_{i}\right\}}_{n}}$ are obtained by solving Equation (26) with Equation (28), which also satisfy the condition in Equation (27).

After performing HOGSVD of Equation (18) to obtain the left and right singular vectors of $R_{\left\{f_{i},f_{o}\right\}}R_{\left\{f_{o},f_{o}\right\}}^{H}$, the transformation matrices $T_{{\left\{f_{i}\right\}}_{\mathrm{MOP}}}$ can be assembled as follows:

(29) $$\left[\begin{array}{c} T_{{\left\{f_{1}\right\}}_{\mathrm{MOP}}}\\ T_{{\left\{f_{2}\right\}}_{\mathrm{MOP}}}\\ \vdots \\ T_{{\left\{f_{P}\right\}}_{\mathrm{MOP}}} \end{array}\right]=V_{e_{s}}\left[\begin{array}{c} U_{e{\left\{f_{1}\right\}}_{s}}^{†}\\ U_{e{\left\{f_{2}\right\}}_{s}}^{†}\\ \vdots \\ U_{e{\left\{f_{P}\right\}}_{s}}^{†} \end{array}\right].$$

Note that since orthonormal columns have not yet been assumed on the matrix $U_{\psi \left\{f\right\}}$ in Theorem 1, the transformation procedure via HOGSVD is still compatible with Theorem 1 without requiring any modifications (For details, see Equations (A13) and (A14) in Appendix A).

We now consider the computational complexity of HOGSVD. It is not surprising that HOGSVD has a heavy computational burden; that is because matrix inversions are intensively used in Equation (20). To avoid the computational burden caused by the matrix inversions, Equation (20) is reformulated by the following technique [51]. It begins by performing the economy-sized QR decomposition of Equation (19);

(30) $$\left[\begin{array}{c} E_{\left\{f_{1}\right\}}\\ E_{\left\{f_{2}\right\}}\\ \vdots \\ E_{\left\{f_{P}\right\}} \end{array}\right]=\left[\begin{array}{c} Q_{\varsigma_{1}}\\ Q_{\varsigma_{2}}\\ \vdots \\ Q_{\varsigma_{P}} \end{array}\right]R_{\varsigma_{}},$$

where $R_{\varsigma_{i}}\in C^{M\times M}$ is the upper triangular matrix, and $Q_{\varsigma_{i}}\in C^{M\times M}$ is a one portion of the $\left(M\times P\right)$-by-*M* matrix resulting from the QR decomposition of Equation (19). Next, S is simplified as

(31) $$S_{\varsigma_{}}=\frac{1}{P\left(P-1\right)}\left(D_{\varsigma_{}}-PI_{M}\right),$$

where

(32) $$D_{\varsigma_{}}=\sum_{i=1}^{P}{\left(Q_{\varsigma_{i}}Q_{\varsigma_{i}}\right)}^{-1}.$$

Performing EVD of Equation (32), then we have $D_{\varsigma_{}}=Z_{\varsigma_{}}\Lambda_{\varsigma_{}}Z_{\varsigma_{}}$, where $Z_{\varsigma_{}}\in C^{M\times M}$ and $\Lambda_{\varsigma_{}}\in R^{M\times M}$ are the matrix of eigenvectors and matrix of eigenvalues, respectively. Finally, the alternative computation of $V_{e}$ is expressed as $R_{\varsigma_{}}Z_{\varsigma_{}}$, where the *K* smallest eigenvalues of $D_{\varsigma_{}}$ are belonged to signal subspace.

Computational complexity of conventional HOGSVD in Equation (20) and optimized HOGSVD in Equation (32) are investigated by applying the following scenario: an M×M matrix addition, subtraction, multiplication and element-wise multiplication follow the traditional way, whereas an M×M matrix inversion and QR decomposition of PM×M matrix are implemented by using Gauss–Jordan elimination algorithm and Householder transformation, respectively. Comparing computation costs of Equations (20) and (32) from Table 1 and Table 2, it is clearly seen that the technique in Equations (31) and (32) simplifies the mathematical model, reduces the matrix operations and improves the speed of $V_{e}$ computation. When $P=M^{i}\;\forall i:i>0$, the optimized HOGSVD has arithmetic complexity of $O\left(M^{i+3}\right)$, which exhibits remarkably less computational complexity than the conventional HOGSVD that is presented as $O\left(M^{2i+3}\right)$. Since *P* in most cases is much greater than *M*, therefore, the cost of the optimized HOGSVD can logically be less than the conventional HOGSVD.

### 3.3. DOA Estimation Scheme

After the transformation matrices are formed by using HOGSVD, we now proceed to describe a framework for estimating the wideband DOAs. We start by simplifying the wideband cross-correlation matrix in Equation (8) with EVD form and substituting with $T_{{\left\{f_{i}\right\}}_{\mathrm{MOP}}}$, as follows:

(33) $$\begin{array}{cc} \frac{1}{P}\sum_{i=1}^{P} & T_{{\left\{f_{i}\right\}}_{\mathrm{MOP}}}R_{\left\{f_{i},f_{i}\right\}}T_{{\left\{f_{i}\right\}}_{\mathrm{MOP}}}^{H}\\ & =\frac{1}{P}\sum_{i=1}^{P}\left(V_{e_{s}}U_{e{\left\{f_{i}\right\}}_{s}}^{†}\right)\left(Q_{{\left\{f_{i}\right\}}_{s}}\Lambda_{{\left\{f_{i}\right\}}_{s}}Q_{{\left\{f_{i}\right\}}_{s}}^{H}+Q_{{\left\{f_{i}\right\}}_{n}}\Lambda_{{\left\{f_{i}\right\}}_{n}}Q_{{\left\{f_{i}\right\}}_{n}}^{H}\right){\left(V_{e_{s}}U_{e{\left\{f_{i}\right\}}_{s}}^{†}\right)}^{H}\\ & =Q\Lambda Q^{H}+\Pi , \end{array}$$

where

(34) $$\begin{array}{cc} \Lambda & =L^{H}\left(\frac{1}{P}\sum_{i=1}^{P}\left(U_{e{\left\{f_{i}\right\}}_{s}}^{†}Q_{{\left\{f_{i}\right\}}_{s}}\right)\Lambda_{{\left\{f_{i}\right\}}_{s}}{\left(U_{e{\left\{f_{i}\right\}}_{s}}^{†}Q_{{\left\{f_{i}\right\}}_{s}}\right)}^{H}\right)L,\\ \Pi & =V_{e_{s}}\left(\frac{1}{P}\sum_{i=1}^{P}\left(U_{e{\left\{f_{i}\right\}}_{s}}^{†}Q_{{\left\{f_{i}\right\}}_{n}}\right)\Lambda_{{\left\{f_{i}\right\}}_{n}}{\left(U_{e{\left\{f_{i}\right\}}_{s}}^{†}Q_{{\left\{f_{i}\right\}}_{n}}\right)}^{H}\right)V_{e_{s}}^{H}, \end{array}\;Q=V_{e_{s}}L.$$

Here, $\Lambda \in C^{K\times K}$ and $Q\in C^{M\times K}$ are the diagonal matrix of eigenvalues and matrix of eigenvectors of Equation (33) in signal subspace, and $L\in C^{K\times K}$ possess unitary property by the fact that $Q,V_{e_{s}}$ are the matrices with orthonormal columns [46,47]. Remark that $R_{\left\{f_{i},f_{i}\right\}}$ is also derived by performing EVD; the matrices $Q_{{\left\{f_{i}\right\}}_{s}}\in C^{M\times K}$, $\Lambda_{{\left\{f_{i}\right\}}_{s}}\in R^{K\times K}$ are the eigenvectors and diagonal matrix of eigenvalues in signal subspace, and likewise, $Q_{{\left\{f_{i}\right\}}_{n}}\in C^{M\times M-K}$, $\Lambda_{{\left\{f_{i}\right\}}_{n}}\in R^{M-K\times M-K}$ are with noise subspace. Furthermore, considering only the signal subspace by focusing on the *K* largest singular values Λ, we can expect that Equation (33) is equivalent to Equation (8);

(35) $$\begin{array}{cc} Q\Lambda Q^{H} & \equiv A\left(\phi ,\theta ,f_{o}\right)\left(\frac{1}{P}\sum_{i=1}^{P}S_{\left\{f_{i},f_{i}\right\}}\right)A^{H}\left(\phi ,\theta ,f_{o}\right), \end{array}$$

which can be proved by employing Lemma 1, Equations (12)–(14), and Equations (A4)–(A6) on Appendix A (We omit the proof since the result is easily obtained by performing straightforward substitution). In this state, $T_{{\left\{f\right\}}_{\mathrm{MOP}}}$ provides an efficient way to transform any given *f* into $f_{o}$ by observing the solution across frequency bands without loss of generality; it means that the transformation is no longer biased by the pair of distinct frequencies $\left\{f,f_{o}\right\}$. Furthermore, it is clearly seen that the wideband cross-correlation matrix in Equation (33) is the combination of narrowband sample cross-correlation matrices across all frequency bins, but its array manifolds are focused on the single reference frequency by using $T_{{\left\{f\right\}}_{\mathrm{MOP}}}$, which is now feasible to estimate the wideband DOAs by employing any recent subspace-based technique for estimating narrowband DOAs [18,20,21,22,23,24,25,26], but using this wideband correlation matrix instead of the narrowband correlation matrix. Practical examples, such as MUSIC and ESPRIT, will be presented to showcase its applicability and effectiveness in the next section.

Remarks: In case of the L-shaped array structure in Equation (2), we can repeat the proposed transformation procedure to find the solution for *x* subarray in Equation (2) and (3); starting from Equation (7) by replacing $r\left(t,f\right)$ with $x\left(t,f\right)$, the solution for the *x* subarray can be given by:

(36) $$T_{x{\left\{f_{i}\right\}}_{\mathrm{MOP}}}=V_{x,e_{s}}U_{x,e{\left\{f_{i}\right\}}_{s}}^{†},$$

(37) $$\frac{1}{P}\sum_{i=1}^{P}T_{x{\left\{f_{i}\right\}}_{\mathrm{MOP}}}R_{x\left\{f_{i},f_{i}\right\}}T_{x{\left\{f_{i}\right\}}_{\mathrm{MOP}}}^{H}=Q_{x}\Lambda_{x}Q_{x}^{H}+\Pi_{x},$$

(38) $$Q_{x}\Lambda_{x}Q_{x}^{H}\equiv A_{x}\left(\phi ,f_{o}\right)\left(\frac{1}{P}\sum_{i=1}^{P}S_{\left\{f_{i},f_{i}\right\}}\right)A_{x}^{H}\left(\phi ,f_{o}\right).$$

By performing the same procedure, the solution for *z* subarray is likewise given by replacing $x\left(t,f\right),A_{x}\left(\phi ,f_{o}\right)$ with $z\left(t,f\right),A_{z}\left(\theta ,f_{o}\right)$ and the subscript *x* with *z* in Equations (36)–(38);

(39) $$T_{z{\left\{f_{i}\right\}}_{\mathrm{MOP}}}=V_{z,e_{s}}U_{z,e{\left\{f_{i}\right\}}_{s}}^{†},$$

(40) $$\frac{1}{P}\sum_{i=1}^{P}T_{z{\left\{f_{i}\right\}}_{\mathrm{MOP}}}R_{z\left\{f_{i},f_{i}\right\}}T_{z{\left\{f_{i}\right\}}_{\mathrm{MOP}}}^{H}=Q_{z}\Lambda_{z}Q_{z}^{H}+\Pi_{z},$$

(41) $$Q_{z}\Lambda_{z}Q_{z}^{H}\equiv A_{z}\left(\theta ,f_{o}\right)\left(\frac{1}{P}\sum_{i=1}^{P}S_{\left\{f_{i},f_{i}\right\}}\right)A_{z}^{H}\left(\theta ,f_{o}\right).$$

#### 3.3.1. DOA Estimation Scheme via MUSIC

MUSIC estimates the DOA of the sources by locating the peaks of MUSIC spectrum along with exploiting the orthogonality of the signal and noise subspaces [12,48]. Let us define the complementary orthogonal space $\left(I_{M}-QQ^{H}\right)$ which is orthogonal to $A\left(\phi ,\theta ,f_{o}\right)$;

(42) $$a^{H}\left(\phi_{k},\theta_{k},f_{o}\right)\left(I_{M}-QQ^{H}\right)a\left(\phi_{k},\theta_{k},f_{o}\right)=0,$$

for all $k\in \left\{1,2,\cdots ,K\right\}$, where $a\left(\phi_{k},\theta_{k},f_{o}\right)\in C^{M}$ is a $k^{\mathrm{th}}$ column of $A\left(\phi ,\theta ,f_{o}\right)$ as shown in Equation (3). Additionally, the following complementary orthogonal space is also valid;

(43) $$a^{H}\left(\phi_{k},\theta_{k},f_{o}\right)\left(I_{M}-V_{e_{s}}V_{e_{s}}^{H}\right)a\left(\phi_{k},\theta_{k},f_{o}\right)=0,$$

by the fact that $QQ^{H}=V_{e_{s}}\left(LL^{H}\right)V_{e_{s}}^{H}=V_{e_{s}}V_{e_{s}}^{H}$, which implies that it is possible to reduce a computational complexity of Equation (33) by using only $V_{e_{s}}$ instead of calculating Q. The computationally efficient two-dimensional MUSIC (2D-MUSIC) spectrum is expressed as

(44) $$p_{{}_{2D-\mathrm{MUSIC}}}\left(\phi ,\theta \right)=\frac{1}{a^{H}\left(\phi ,\theta ,f_{o}\right)\left(I_{M}-V_{e_{s}}V_{e_{s}}^{H}\right)a\left(\phi ,\theta ,f_{o}\right)}.$$

When the denominator in Equation (44) approaches zero for the true angles of the signals, the 2D-MUSIC spectrum will have peak spikes indicating this angles. In case of the L-shaped array structure, the *x* and *z* subarray angles are estimated separately by locating the spectral peaks of the following equations:

(45) $$\begin{array}{cc} p_{x_{\mathrm{MUSIC}}}\left(\phi \right) & =\frac{1}{a_{x}^{H}\left(\phi ,f_{o}\right)\left(I_{N}-V_{x,e_{s}}V_{x,e_{s}}^{H}\right)a_{x}\left(\phi ,f_{o}\right)},\\ p_{z_{\mathrm{MUSIC}}}\left(\theta \right) & =\frac{1}{a_{z}^{H}\left(\theta ,f_{o}\right)\left(I_{N}-V_{z,e_{s}}V_{z,e_{s}}^{H}\right)a_{z}\left(\theta ,f_{o}\right)}, \end{array}$$

where $a_{x}\left(\phi ,f_{o}\right),a_{z}\left(\theta ,f_{o}\right)\in C^{N}$ are $i^{\mathrm{th}}$ column of $A_{x}\left(\phi ,f_{o}\right),A_{z}\left(\theta ,f_{o}\right)$, respectively.

#### 3.3.2. DOA Estimation Scheme via ESPRIT

We start by recalling the array manifold $A_{x}\left(\phi ,f_{o}\right)$ and $A_{z}\left(\theta ,f_{o}\right)$ in Equation (3). ESPRIT takes advantage of the rotational invariance property of ULA [13], as follows:

(46) $$\begin{array}{cc} A_{x_{2}}\left(\phi ,f_{o}\right) & =A_{x_{1}}\left(\phi ,f_{o}\right)\Phi_{x},\\ A_{z_{2}}\left(\theta ,f_{o}\right) & =A_{z_{1}}\left(\theta ,f_{o}\right)\Theta_{z}, \end{array}$$

where

(47) $$\begin{array}{cc} \Phi_{x} & =\mathrm{diag}\left(e^{\alpha_{x}\left(\phi_{1},f_{o}\right)j},e^{\alpha_{x}\left(\phi_{2},f_{o}\right)j},\cdots ,e^{\alpha_{x}\left(\phi_{K},f_{o}\right)j}\right),\\ \Theta_{z} & =\mathrm{diag}\left(e^{\alpha_{z}\left(\theta_{1},f_{o}\right)j},e^{\alpha_{z}\left(\theta_{2},f_{o}\right)j},\cdots ,e^{\alpha_{z}\left(\theta_{K},f_{o}\right)j}\right), \end{array}$$

$A_{x_{1}}\left(\phi ,f_{o}\right),A_{z_{1}}\left(\theta ,f_{o}\right)\in C^{N-1\times K}$ and $A_{x_{2}}\left(\phi ,f_{o}\right),A_{z_{2}}\left(\theta ,f_{o}\right)\in C^{N-1\times K}$ stand for the first and last $\left(N-1\right)$ rows of $A_{x}\left(\phi ,f_{o}\right),A_{z}\left(\theta ,f_{o}\right)$, respectively. Similar to [20,21,26], the matrices $Q_{x},Q_{z}$ can be simplified with Equations (3), (36)–(38) and (46), as follows:

(48) $$\begin{array}{cc} Q_{x_{1}} & =A_{x_{1}}\left(\phi ,f_{o}\right)C_{x}^{-1},\\ Q_{x_{2}} & =A_{x_{2}}\left(\phi ,f_{o}\right)C_{x}^{-1}, \end{array}\;\;\begin{array}{cc} Q_{z_{1}} & =A_{z_{1}}\left(\theta ,f_{o}\right)C_{z}^{-1},\\ Q_{z_{2}} & =A_{z_{2}}\left(\theta ,f_{o}\right)C_{z}^{-1}, \end{array}$$

where $C_{x},C_{z}\in C^{K\times K}$ are invertible matrices, $Q_{x_{1}},Q_{z_{1}}\in C^{N-1\times K}$ and $Q_{x_{2}},Q_{z_{2}}\in C^{N-1\times K}$ stand for the first and last $\left(N-1\right)$ rows of $Q_{x},Q_{z}$, respectively. Considering Equation (48), we can construct new matrices $\Gamma_{x},\Gamma_{z}$ as follows:

(49) $$\begin{array}{cc} \Gamma_{x} & =Q_{x_{1}}^{†}Q_{x_{2}}\\ & =C_{x}\Phi_{x}C_{x}^{-1}, \end{array}\;\;\begin{array}{cc} \Gamma_{z} & =Q_{z_{1}}^{†}Q_{z_{2}}\\ & =C_{z}\Theta_{z}C_{z}^{-1}. \end{array}$$

The angles $\phi_{k},\theta_{k}$ can thus be estimated by the eigenvalues of $\Gamma_{x},\Gamma_{z}$, as follows:

(50) $$\phi_{k}=\cos^{-1}\left(\mathrm{angle}\left(\lambda_{x_{k}}\right)\frac{\lambda }{2\pi d}\right),\;\;\theta_{k}=\cos^{-1}\left(\mathrm{angle}\left(\lambda_{z_{k}}\right)\frac{\lambda }{2\pi d}\right),$$

where $\lambda_{x_{k}},\lambda_{z_{k}}\in C$ is the $k^{\mathrm{th}}$ eigenvalue of $\Gamma_{x},\Gamma_{z}$, respectively. Furthermore, it is possible to reduce the computational complexity by using only $V_{e_{s}}$ as well as MUSIC;

(51) $$\begin{array}{cc} V_{x_{1},e_{s}}^{†}V_{x_{2},e_{s}} & =L_{x}\Gamma_{x}L_{x}^{-1}\\ & =\left(L_{x}C_{x}\right)\Phi_{x}{\left(L_{x}C_{x}\right)}^{-1}, \end{array}\;\;\begin{array}{cc} V_{z_{1},e_{s}}^{†}V_{z_{2},e_{s}} & =L_{z}\Gamma_{z}L_{z}^{-1}\\ & =\left(L_{z}C_{z}\right)\Theta_{z}{\left(L_{z}C_{z}\right)}^{-1}, \end{array}$$

where

(52) $$\begin{array}{cc} V_{x_{1},e_{s}} & =A_{x_{1}}\left(\phi ,f_{o}\right){\left(L_{x}C_{x}\right)}^{-1},\\ V_{x_{2},e_{s}} & =A_{x_{2}}\left(\phi ,f_{o}\right){\left(L_{x}C_{x}\right)}^{-1}, \end{array}\;\;\begin{array}{cc} V_{z_{1},e_{s}} & =A_{z_{1}}\left(\theta ,f_{o}\right){\left(L_{z}C_{z}\right)}^{-1},\\ V_{z_{2},e_{s}} & =A_{z_{2}}\left(\theta ,f_{o}\right){\left(L_{z}C_{z}\right)}^{-1}, \end{array}$$

$V_{x_{1},e_{s}},V_{z_{1},e_{s}}\in C^{N-1\times K}$ and $V_{x_{2},e_{s}},V_{z_{2},e_{s}}\in C^{N-1\times K}$ stand for the first and last $\left(N-1\right)$ rows of $V_{x,e_{s}},V_{z,e_{s}}$, respectively.

## 4. Numerical Simulations

In this section, performances of fusion methods by using the proposed framework are demonstrated in four types of the following scenarios: (1) a performance of selected method and the proposed methods with respect to source types, (2) the performance with respect to the number of microphone elements, (3) the performance with considering automatic pairing of the *x* and *z* subarray angles, and (4) the performance under a reverberation environment. Scenarios 1, 2 and 4 have to find DOA of *x* and *z* subarray angles separately by using the data model in Equation (2). Whereas Scenario 3 has to find DOA of *x* and *z* subarray angles simultaneously with considering automatic pairing, by using the data model in Equation (1). We provided the simulation tests of the proposed methods in comparison to following methods: IMUSIC [31], TOFS [32], TOPS [33], Squared-TOPS [34], WS-TOPS [35]. Note that the CSS-based methods are excluded in these tests; this is because unintended biases, causing by a process of DOA preliminary estimation, should be taken into consideration to other candidate methods as discussed in the literature [31,32,33,41].

To measure the overall performance of estimating the *x* and *z* subarray angles for each scenario, the root-mean-square-error (RMSE) and standard division (SD) are defined as the following equations;

(53) $$\mathrm{RMSE}=\sqrt{\frac{1}{2JK}\sum_{j=1}^{J}\sum_{k=1}^{K}\left({\left({\hat{\phi }}_{k}^{\left(j\right)}-\phi_{k}\right)}^{2}+{\left({\hat{\theta }}_{k}^{\left(j\right)}-\theta_{k}\right)}^{2}\right)},$$

(54) $$\mathrm{SD}=\sqrt{\frac{1}{2JK}\sum_{j=1}^{J}\sum_{k=1}^{K}\left({\left({\hat{\phi }}_{k}^{\left(j\right)}-{\bar{\phi }}_{k}\right)}^{2}+{\left({\hat{\theta }}_{k}^{\left(j\right)}-{\bar{\theta }}_{k}\right)}^{2}\right)},$$

where *K* is the source number, *J* is the number of trials, ${\hat{\phi }}_{k}^{\left(j\right)},{\hat{\theta }}_{k}^{\left(j\right)}$ represent the estimated *x* and *z* subarray angles each trial, ${\bar{\phi }}_{k},{\bar{\theta }}_{k}$ represent an average of the estimated *x* and *z* subarray angles, and $\phi_{k},\theta_{k}$ represent true *x* and *z* subarray angles.

Computer simulations were carried out in Matlab^®^ R2017a, using PC with Debian GNU/Linux 9.4 × 86_64, Intel^®^ Core^™^ i5-4590 CPU 3.30 GHz, 16G RAM, Intel^®^ Math Kernel Library 11.3.1 on BLAS and LAPACK 3.5.0. Each scenario is repeated 100 times, and simulation parameters are chosen as follows: sampling frequency is 48 kHz, an output of each microphone is captured at 1 s, speed of sound *c* is 343 m/s, the spacing of microphone elements *d* is 5 cm, STFT focusing frequency range is from 0.1 to 16 kHz, the reference frequency $f_{o}$ is 3.43 kHz. Note that we used perturbations of the true angles by adding Gaussian random noise.

### 4.1. Scenario 1: Performance with Respect to Source Types

**Figure 2:**
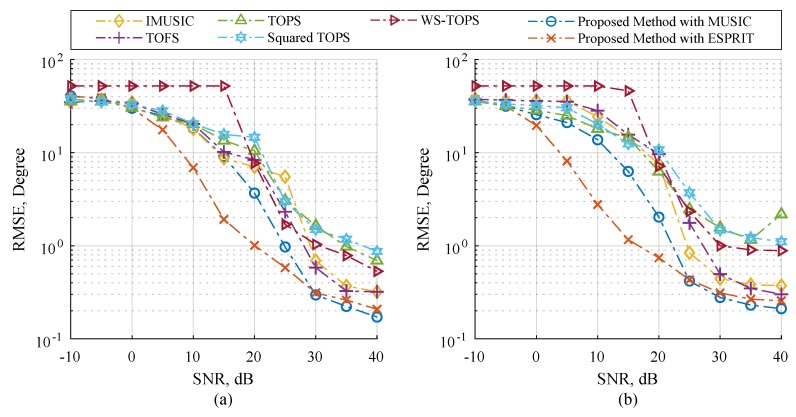
RMSE estimation performance versus SNR on Scenario 1; (**a**) three different human speeches, and (**b**) three uncorrelated musical sounds where six microphones are employed each subarray.

**Figure 3:**
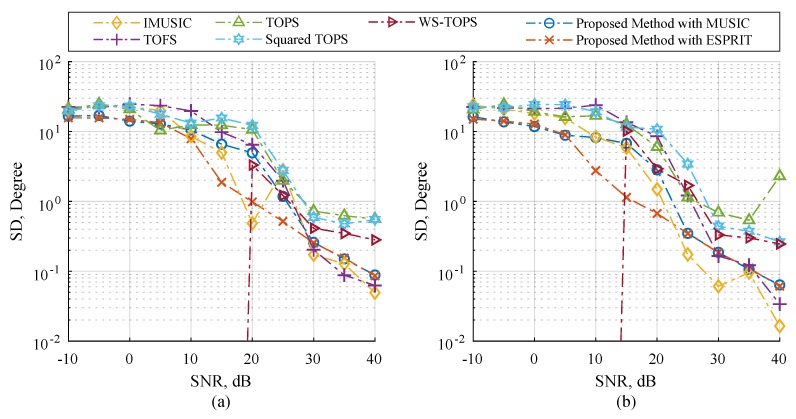
SD estimation performance versus SNR on Scenario 1; (**a**) three different human speeches, and (**b**) three uncorrelated musical sounds where six microphones is employed each subarray.

Figure 2 and Figure 3 showed performance comparisons of the selected methods and the proposed methods in term of RMSE and SD over a range of SNR. The proposed methods are the modified MUSIC in Equation (45) and ESPRIT in Equations (50)–(52). The number of microphone elements each subarray is six, and the three uncorrelated source angles $\left(\phi_{k},\theta_{k}\right)$ are placed at $\left(41.41^{\circ },60^{\circ }\right)$, $\left(60^{\circ },45^{\circ }\right)$ and $\left(75.52^{\circ },30^{\circ }\right)$. In Figure 2a and Figure 3a, sources are human speeches. Sources in Figure 2b and Figure 3b are recorded sound on a piano comprising various monochromatic notes and containing sampling frequency range up to 48 kHz. Note that all sources are not stationary signals. The results in Figure 2 and Figure 3 showed that the proposed method with ESPRIT can efficiently handle both source types compared to other candidate methods with acceptable SNR ranges. Subsequently, it is interesting to take a close look at 40 dB SNR in Figure 2 and Figure 3 where IMUSIC, TOFS, the proposed method with MUSIC and ESPRIT showed very low RMSE, which could attest to good DOA estimation. When decreasing the SNR to 25 dB, IMUSIC and TOFS begin to demonstrate worse RMSE quality, which is much higher than the proposed methods, and it is clearly seen when decreasing the SNR to 10 dB that all tested methods are significantly dominated, but the proposed method with ESPRIT is still associated with more satisfactory results compared to using other methods. It should be mentioned that IMUSIC and TOFS require the number of sensor elements to be much higher than the number of sources to achieve fairly good results [31,32,33,41]. Hence, the simulation results in Figure 2 and Figure 3 are able to provide evidence that the proposed methods perform better in estimation than other candidate methods when the incident sources are wideband and non-stationary signals. Although the performances of the proposed method with MUSIC is also dominated by the noises, the overall performances is still more effective than other methods.

### 4.2. Scenario 2: Performance with Respect to the Number of Microphone Elements

**Figure 4:**
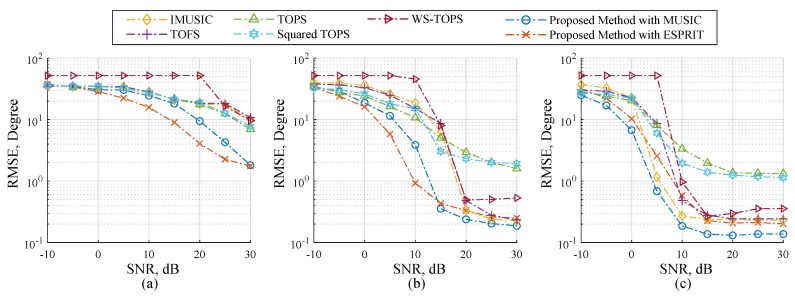
RMSE estimation performance versus SNR on Scenario 2; three human speeches are employed and the number of microphone elements each subarray on (**a**) N=4, (**b**) N=8, and (**c**) N=12.

**Figure 5:**
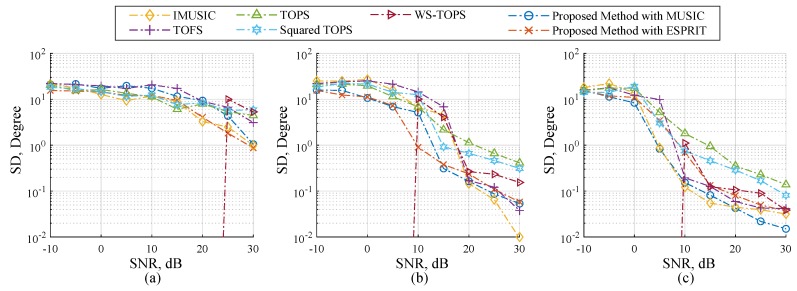
SD estimation performance versus SNR on Scenario 2; three human speeches are employed and the number of microphone elements each subarray on (**a**) N=4, (**b**) N=8, and (**c**) N=12.

Figure 4 and Figure 5 illustrates performance comparisons of the selected methods and the proposed methods in term of RMSE and SD over a range of SNR. The three uncorrelated source angles are human speeches, and are placed as previously used. Firstly, let us start by looking at the case of twelve microphones in Figure 4c and Figure 5c. IMUSIC, TOFS and WS-TOPS exhibited remarkably low levels of RMSE in the SNR range from 15 to 30 dB; this is because their performances dramatically depend on the number of sensor elements more than the number of sources [31,32,33,41]. Likewise, the proposed method with MUSIC and ESPRIT also demonstrated very low RMSE, which may imply that the performance of the proposed methods, IMUSIC, TOFS and WS-TOPS are especially effective for a wideband DOA estimation. However, the low number of microphone elements should be considered for providing more practical applications. In the case of eight microphones in each subarray, the performances of the selected methods are dominated by the number of microphone elements as illustrated in Figure 4b and Figure 5b. Furthermore, the performances of selected methods are dramatically degraded when employing four microphones as illustrated in Figure 4a and Figure 5a. The relevant reason is that an undesirable false peak in the spatial spectrum of the selected methods occurred, caused by the perturbation of noise; when power of the noise at some frequency is high or grater than source power, the orthogonality between the noise subspace and search space at that frequency may be not sufficient to prevent the false-alarm peaks [41]. On the contrary, RMSE performance of the proposed methods are also dominated, but less than the other methods, by exhibiting the subspace for all frequency bins simultaneously as shown in Section 3. Therefore, the proposed methods provide substantially better RMSE performance than the other methods, which implies that dependency between the number of microphone elements and sources can be relaxed. This substantial ability is more meaningful for many practical applications.

### 4.3. Scenario 3: Performance with Considering Automatic Pairing

**Figure 6:**
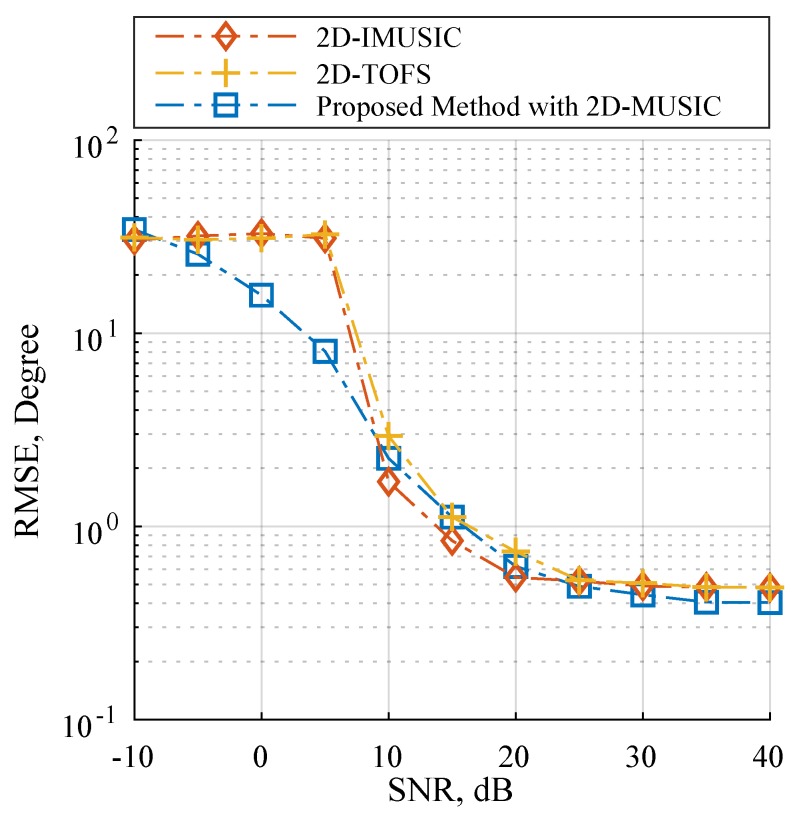
RMSE estimation performance versus SNR on Scenario 3 where M=8.

**Figure 7:**
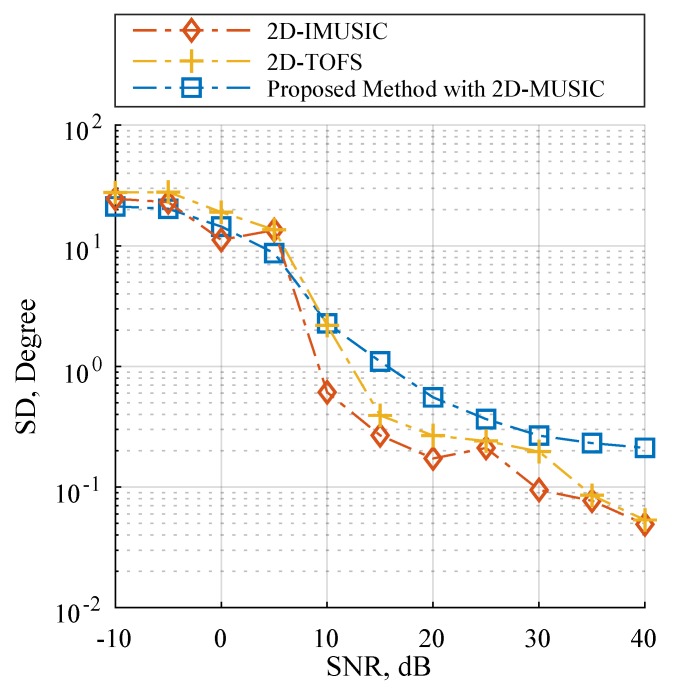
SD estimation performance versus SNR on Scenario 3 where M=8.

This scenario estimated the DOA of *x* and *z* subarray angles simultaneously with considering automatic pairing and following the data model in Equation (1). As the L-shaped array structure consisting of two ULAs as illustrated in Figure 1, some research works estimate the DOA of *x* and *z* subarray angles separately by implementing 1D DOA estimation for each ULA [17,18,19,20,21,22,23,24,25,26]. When utilizing more than one source, these algorithms require an additional angle pair matching procedure to map the relationship between the two independent subarray angles. For instance, finding the corresponding angle pairs by rearranging the alignment of $a_{x}\left(\phi_{k},f\right)$ with a fixed right-hand side of the array manifolds of the *z*-subarray in the sample cross-covariance matrix [52]. It should be noted that a pair-matching procedure may results in a performance degradation caused by pair-matching error. In order to achieve the automatic pairing without the pair-matching procedure, we selected the modified 2D-MUSIC in Equation (44) as the proposed method in this scenario. Furthermore, TOPS, Squared-TOPS, WS-TOPS are excluded in these tests by the fact that the methods have only supported the ULA model. Note that the 2D peak finding algorithm was employed on 2D-IMUSIC, 2D-TOFS and the proposed method. Figure 6 and Figure 7 showed performance comparisons of 2D-IMUSIC, 2D-TOFS and the proposed method in term of RMSE and SD over a range of SNR, where the number of microphone elements including all subarray is eight, the three uncorrelated source angles are human speeches, and are placed as previously used. Figure 6 indicates that the proposed method with 2D-MUSIC exhibits extremely similar overall performances to 2D-IMUSIC and 2D-TOFS when the SNR increases to more than 10 dB; however, computational burden of the proposed method can be significantly lower than those of the other methods, which Section 4.5 will reveal further insight.

### 4.4. Scenario 4: Performance under Reverberation Environment

**Figure 8:**
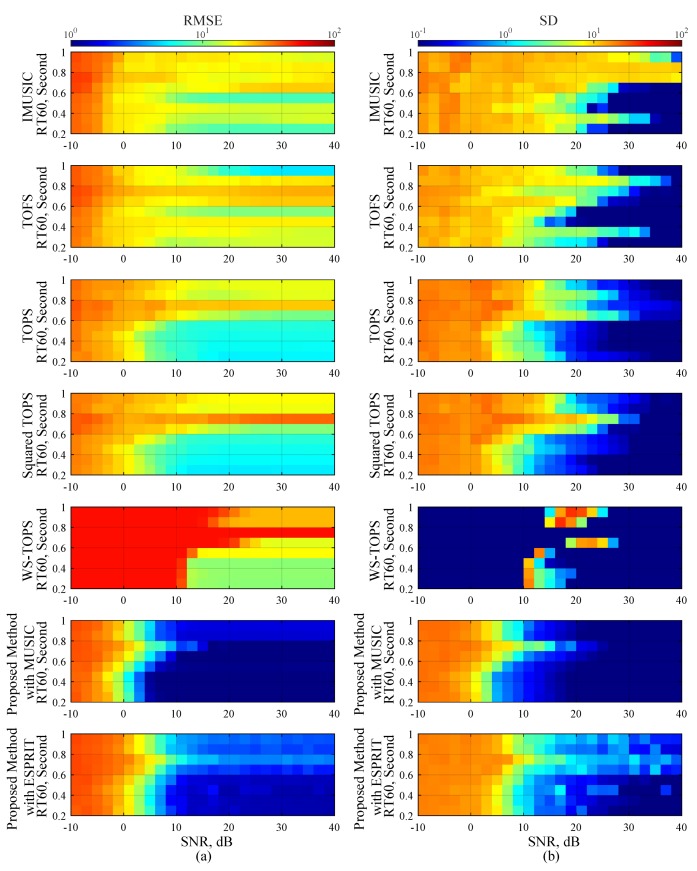
Performance evaluations of Scenario 4; (**a**) RMSE estimation performance versus SNR, and (**b**) SD estimation performance versus SNR, where three uncorrelated human speeches are employed along with a reverberant environment. The reverberations were simulated by the following procedure [53], where dimensions of enclosure room is 15×15×5 m, a measurement protocol of reverberation time is RT60, and wall absorption coefficients are followed on Table 3.

In this scenario, we compared RMSE and SD performances of the proposed methods to other methods with respect to reverberation time. This scenario estimated DOA of *x* and *z* subarray angles separately by using the data model in Equation (2) without considering automatic pairing. The proposed methods in this scenario are the modified MUSIC in Equation (45) and ESPRIT on Equations (50)–(52). The reverberations were simulated by the following procedure [53], and its simulated wall absorption coefficients are shown in Table 3, where the dimensions of enclosure room is 15×15×5 m, a measurement protocol of reverberation time is RT60, and the reverberation time is from 200 to 1000 ms. The three uncorrelated source angles are employed in the same way as previously used, and the number of microphone elements in each subarray is twelve. Figure 8 illustrated performance comparisons of the selected methods and the proposed methods, where a color of the graph on Figure 8a denotes RMSE, whereas a color of the graph on Figure 8b denotes SD estimation performance. The vertical axis is represented as the reverberation time and horizontal axis is represented as a range of SNR. Simulation results in Figure 8 indicated that reverberation has strong effects on RMSE and SD performances in both of the selected methods and the proposed methods, and the performances decreased more significantly at the high noise levels and the long reverberation times. Since the reverberation time is decreasing, all selected methods begin to demonstrate low RMSE. It means that the trade-off between the robustness of reverberation and SNR should be considered deeply in actual applications, for instance, applying a reverberation cancellation technique or a noise cancellation technique to provide much more reliable estimation performances of both RMSE and SD. The proposed methods, however, largely outperform the other methods with respect to the reverberation time index and SNR level range between 10 and 40 dB without considering the trade-off. This can support that the performance of the proposed methods can be especially effective for a wideband DOA estimation under a reverberant environment.

### 4.5. Computational Complexity

**Figure 9:**
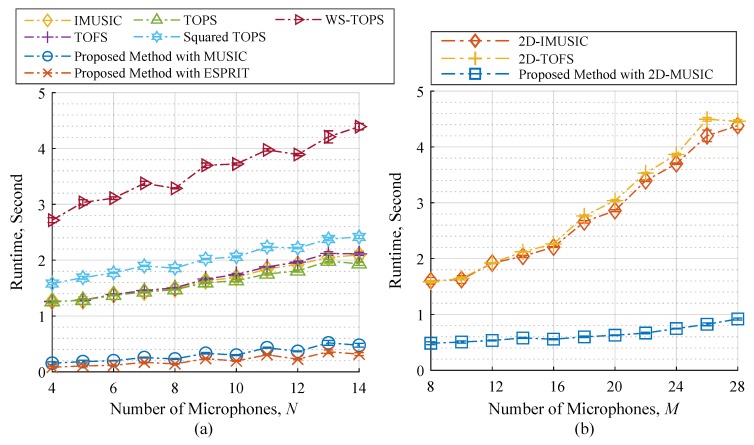
Computational complexities; (**a**) changing the number of microphone elements each subarray *N*, and (**b**) the number of microphone elements including all subarray *M* where the number of incident sources K=3.

Computational complexity of the proposed methods was evaluated using execution time measurement under a stable environment. We provided a computational complexity in comparison with the following cases: (1) calculating DOAs of *x* and *z* subarray angles separately as shown in Figure 9a, and (2) calculating the DOAs of both subarray angles simultaneously as shown in Figure 9b. Note that computational burdens of a peak searching algorithm are relevant in this study, where the number of searching angle in each subarray is 180. It is apparently seen in Figure 9 that computation time of the other methods presented higher growth rates than the proposed methods. This is because the peak searching algorithm execution time is potentially high, and almost all selected methods require intensive computations by testing the orthogonality of subspace and search space of narrowband sample cross-correlation matrices for all frequency bins, which results in high computation costs. On the contrary, the proposed methods transform all narrowband sample cross-correlation matrices across all frequency bins into a single matrix as shown in Equations (33)–(35), and this matrix contains useful information of source cross-correlation matrices across all frequency bins as $\frac{1}{P}\sum_{i=1}^{P}S_{\left\{f_{i},f_{i}\right\}}$; in other words, the orthogonality testing of subspace and search space can be done by using the wideband cross-correlation matrix in Equations (33)–(35) instead of narrowband sample cross-correlation matrices for all frequency bins. Therefore, the computational complexity of the proposed methods remarkably less than the other methods, which is confirmed by the test results in Figure 9.

## 5. Experimental Results

**Figure 10:**
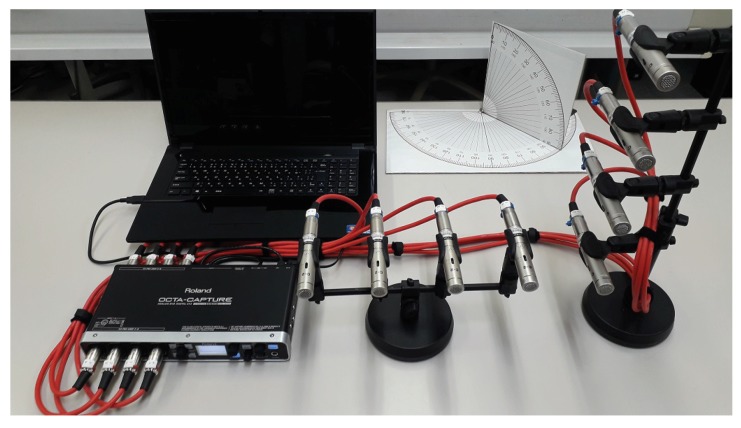
Photograph of the microphone array system.

**Figure 11:**
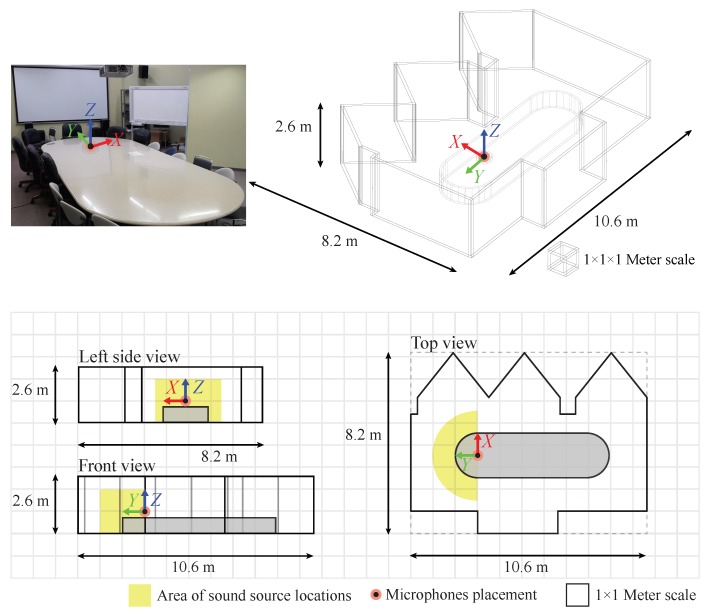
Photograph of the experimental environment, floor plan and the room dimensions.

In this section, experiments were carried out to examine the performance of the proposed methods. Experimental parameters were chosen as the previous simulations, except as follows: We used human speakers as sources of the original speech with random sentences. Their speeches were recorded for 20 runs continuously, and each record signal, approximating 1 min long, was cut into 3 s epochs. Structure of the microphone was followed by Figure 1 and Figure 10, and the specifications of the microphone and its recording device were followed on Table 4. The experiment was performed in an indoor meeting room, and its dimensions are shown in Figure 11, where sound pressure level in the meeting room in a normal situation is 46.6 dBA, and the estimated reverberation time is based on RT60 is 219 ms.

Two scenarios are considered: (1) estimating DOA of *x* and *z* subarray angles separately, and (2) estimating DOA of *x* and *z* subarray angles simultaneously while considering automatic pairing. In case of Experiment 1, the proposed methods are the modified MUSIC in Equation (45) and ESPRIT in Equations (50)–(52), comparing with the following methods: IMUSIC [31], TOFS [32], TOPS [33], Squared-TOPS [34], WS-TOPS [35]. In case of Experiment 2, the proposed method is the modified 2D-MUSIC in Equation (44), comparing with 2D-IMUSIC [31], and 2D-TOFS [32].

Table 5 and Table 6 showed performance comparisons of the selected methods and the proposed method in term of RMSE over the range of source number, where Table 5 is for Experiment 1, and Table 6 is for Experiment 2. The boldfaced results highlight the optimal minimum RMSE in each problem. As highlighted in Table 5, the performance of IMUSIC exhibited the lowest RMSE when a single source was used, but the performance of the other methods including the proposed methods also exhibited similarly low RMSE in an acceptable error range. When the two sources are performed, the performance of TOPS, Squared-TOPS and WS-TOPS are directly dominated, whereas IMUSIC, TOFS and the proposed methods are slightly dominated, but still maintained sufficiently good performance. When the incident sources are increasing to three, we clearly see that the performance of IMUSIC, TOFS, TOPS, Squared-TOPS and WS-TOPS are significantly dominated by the number of incident sources, because those methods require the number of sensor elements to be much more higher than the number of sources to achieve reasonably good results, which can be verified by referring to the simulation results in Section 4 and Figure 4 and Figure 5. The proposed methods, however, are able estimate the DOA of three sources effectively and better than the selected methods. The reason is that the proposed methods focus on the subspace across all frequency bins simultaneously instead of focusing each frequency band individually, which is stated in Section 3.2. In case of Experiment 2 in Table 6, the experiment results indicate that the proposed method with 2D-MUSIC exhibit extremely similar overall performances to 2D-IMUSIC and 2D-TOFS. As already stated in Section 4.5, the computational complexity of the proposed method is definitely lower than 2D-IMUSIC and 2D-TOFS by the fact that those methods check the orthogonality of subspace and search space of narrowband sample cross-correlation matrices for all frequency bins, resulting in very high computation requirement. The proposed method tests the orthogonality of subspace and search space by using the wideband sample cross-correlation matrix in Equation (33) instead of using the subspace of narrowband sample cross-correlation matrices for all frequency bins, but it is sufficient to exhibit significant effects as well as using the subspace of narrowband sample cross-correlation matrices for all frequency bins. In the end, the experimental results from Table 5 and Table 6 are able to provide evidence that the proposed methods have better estimating performance than other methods with respect to the number of incident sources.

Since the sound source directions are static in Table 5 and Table 6, it is necessary to consider moving sound sources for more practical use. In future work, we will extend the proposed method for moving sound sources, and further develop the prototype to support more realistic tasks.

## 6. Conclusions

An efficient framework for estimating DOA of wideband sound sources was presented. The issue of transforming multiple narrowband cross-correlation matrices for all frequency bins into a wideband cross-correlation matrix has been addressed successfully by focusing on signal subspace for all frequency bins simultaneously instead of the pairing of temporal and reference frequency as done by the CSS-based methods. A new solution to this problem has been given by performing HOGSVD of the array of novel cross-correlation matrices, where elements in the row and column positions are a sample cross-correlation matrix between received signal and itself on two distinct frequencies. It was shown in the theoretical analysis that the proposed transformation procedure provided the best solution under appropriate constraints, and no longer required any process of DOA preliminary estimation. Subsequently, we provided an alternative to construct the wideband cross-correlation matrix via the proposed transformation procedure, and wideband DOAs were estimated easily using this wideband matrix along with a single scheme of estimating DOAs in any narrowband subspace methods. A major contribution of this paper is that the proposed framework enables cutting-edge studies in the recent narrowband subspace methods to estimate DOA of the wideband sources directly, which results in reducing computational complexity and facilitating the estimation algorithm. We also have performed several examples of using the proposed framework, such as 2D-MUSIC, MUSIC, and ESPRIT method integration with the L-shaped microphone arrays. Furthermore, the simulation and experimental results showed that the fusion methods by using the proposed framework exhibited especially effective performance compared to other wideband DOA estimation methods over a range of SNR with much fewer sensors, high noise and reverberation conditions. We believe that the proposed method represents an efficient way for wideband DOA estimation and would be able to improve wideband DOA estimates not only for acoustic signal processing but also other possible related fields. 

## Figures and Tables

**Table 1:** Command used in HOGSVD.

Command Name	Command Counts	
	HOGSVD in Equation (20)	Optimized HOGSVD in Equation (32)
Matrix Addition/Subtraction	P(P−1)−1	P−1
Element-wise Multiplication	1	0
Matrix Multiplication	3P(P−1)	$P+{\left\{1\right\}}^{*}$
Matrix Inversion	P(P−1)	P
QR Decomposition	0	1
Eigenvalue Decomposition (EVD)	1	1

Remark: ${\left\{\cdots \right\}}^{*}$ is caused by a matrix multiplication of $R_{\varsigma_{}}Z_{\varsigma_{}}$.

**Table 2:** Computational complexities.

Command Name	Complex Floating Point Operations per Command
Matrix Addition/Subtraction	$M^{2}$
Element-wise Multiplication	$M^{2}$
Matrix Multiplication	$2M^{3}-M^{2}$
Matrix Inversion (Gauss-Jordan elimination)	$\frac{2}{3}M^{3}+\frac{3}{2}M^{2}-\frac{7}{6}M$
QR Decomposition (Householder transformation)	$\left(2P-\frac{2}{3}\right)M^{3}-2PM^{2}+\frac{2}{3}M$
HOGSVD in Equation (20) without counting EVD	$\frac{20P\left(P-1\right)}{3}M^{3}-\frac{P\left(P-1\right)}{2}M^{2}-\frac{7P\left(P-1\right)}{6}M$
Optimized HOGSVD in Equation (32) without counting EVD	$\frac{\left(14P+4\right)}{3}M^{3}-\frac{\left(P+4\right)}{2}M^{2}-\frac{\left(7P-4\right)}{6}M$

**Table 3:** Wall absorption coefficients at various reverberation time in Scenario 4 [53].

Reverberation Time based on RT60 (Millisecond)	Axial Wall Plane					
	Positive Direction			Negative Direction		
	x−z	x−z	x−y	x−z	x−z	x−y
200	0.7236	0.2021	0.6844	0.0792	0.2436	0.5586
300	0.7142	0.1687	0.7666	0.2650	0.2387	0.7043
400	0.7306	0.0555	0.7731	0.4091	0.8493	0.8587
500	0.5064	0.4974	0.8248	0.4189	0.8069	0.7572
600	0.6074	0.6299	0.8028	0.7599	0.6373	0.8209
700	0.7442	0.7624	0.8734	0.6922	0.6480	0.7893
800	0.6779	0.6827	0.7865	0.8045	0.8386	0.8430
900	0.6992	0.7111	0.7741	0.8752	0.8233	0.9081
1000	0.7622	0.7707	0.9394	0.8248	0.8192	0.8398

**Table 4:** System specification

Hardware Type/Parameter	Specification/Value
Audio Interface	Roland^®^ Octa-capture (UA-1010)
Sampling Frequency	48,000 Hz
Microphone Name	Behringer^®^ C-2 studio condenser microphone
Number of Microphones	8
Pickup Patterns	Cardioid (8.9 mV/Pa; 20–20,000 Hz)
Diaphragm Diameter	16 mm
Equivalent Noise Level	19.0 dBA (IEC 651)
SNR Ratio	75 dB
Microphone Structure	L-shaped Array
Spacing of Microphone	9 cm

**Table 5:** Performance evaluation on Experiment 1. The boldfaced results highlight the optimal minimum RMSE.

Incident Sources			RMSE of DOAs (Degree)						
Number	Position	Angle (Degree)	IMUSIC	TOFS	TOPS	Squared TOPS	WS-TOPS	Proposed Method with MUSIC	Proposed Method with ESPRIT
1	$\phi_{1}$	96	0.3050	**0.2050**	1.0950	1.3350	0.5600	0.7750	0.7074
	$\theta_{1}$	86	**0.5400**	1.2600	1.2750	2.0150	0.6850	0.5700	0.6915
		Average	**0.4225**	0.7325	1.1850	1.6750	0.6225	0.6725	0.6995
2	$\phi_{1}$	65	**1.1857**	1.7286	20.0143	28.5857	37.8714	1.5000	2.0284
	$\theta_{1}$	150	9.6000	6.6857	26.3571	39.7857	88.2000	8.8143	**8.6800**
	$\phi_{2}$	55	**1.0714**	1.6857	22.2571	19.4000	32.2429	2.9714	3.8695
	$\theta_{2}$	100	8.3714	8.3857	5.0143	6.7857	60.2286	6.6714	**3.1630**
		Average	5.0571	4.6214	18.4107	23.6393	54.6357	4.9893	**4.4353**
3	$\phi_{1}$	58	**2.1400**	2.3900	46.5500	52.8100	40.9500	3.6600	4.0334
	$\theta_{1}$	55	55.0000	55.0000	55.0000	55.0000	55.0000	9.4300	**4.1057**
	$\phi_{2}$	100	**1.8400**	2.0000	41.5700	62.4000	70.9100	1.8700	2.4554
	$\theta_{2}$	95	95.0000	83.4200	52.4500	71.4800	95.0000	9.7700	**5.8638**
	$\phi_{3}$	130	10.9300	11.8900	28.8300	32.2800	95.2400	8.2500	**6.9071**
	$\theta_{3}$	120	26.9800	25.8400	16.1200	18.0100	91.2800	**5.9400**	7.3165
		Average	31.9817	30.0900	40.0867	48.6633	74.7300	6.4867	**5.1137**

**Table 6:** Performance evaluation on Experiment 2. The boldfaced results highlight the optimal minimum RMSE.

Incident Sources			RMSE of DOAs (Degree)		
Number	Position	Angle (Degree)	2D-IMUSIC	2D-TOFS	Proposed Method with 2D-MUSIC
1	$\phi_{1}$	96	0.9000	0.9000	0.9000
	$\theta_{1}$	86	**0.4000**	1.0500	0.7500
		Average	**0.6500**	0.9750	0.8250
2	$\phi_{1}$	57	**0.9500**	1.1500	1.1000
	$\theta_{1}$	91	**1.0500**	1.8000	1.7000
	$\phi_{2}$	139	**4.9500**	5.2000	5.4500
	$\theta_{2}$	96	3.1500	3.3000	**2.0500**
		Average	**2.5250**	2.8625	2.5750
3	$\phi_{1}$	48	**0.9500**	1.5500	1.9500
	$\theta_{1}$	86	1.4500	**0.8000**	2.4500
	$\phi_{2}$	98	**0.9000**	1.8000	1.1500
	$\theta_{2}$	95	**1.4500**	2.1500	2.6000
	$\phi_{3}$	152	2.7000	**2.4000**	5.9000
	$\theta_{3}$	95	4.5000	3.9000	**1.4500**
		Average	**1.9917**	2.1000	2.5833
4	$\phi_{1}$	100	5.8095	6.5238	**3.2857**
	$\theta_{1}$	94	2.4286	2.6190	**1.6667**
	$\phi_{2}$	51	1.2381	**1.0952**	2.5714
	$\theta_{2}$	95	**0.5714**	0.6667	1.3333
	$\phi_{3}$	134	1.9524	**1.8571**	3.9524
	$\theta_{3}$	103	10.0952	10.2857	**9.2857**
	$\phi_{4}$	153	**7.4762**	7.8095	7.8571
	$\theta_{4}$	89	**4.7143**	**4.7143**	5.3810
		Average	**4.2857**	4.4464	4.4167

## Appendix A. Proof of Theorem 1

This appendix provides a detailed derivation of Theorem 1. We begin by considering the cross-correlation matrices in Equation (7). $R_{\left\{f_{o},f_{o}\right\}}$ can be constructed into the EVD form, which is given by

(A1) $$R_{\left\{f_{o},f_{o}\right\}}=Q_{{\left\{f_{o}\right\}}_{s}}\Lambda_{{\left\{f_{o}\right\}}_{s}}Q_{{\left\{f_{o}\right\}}_{s}}+Q_{{\left\{f_{o}\right\}}_{n}}\Lambda_{{\left\{f_{o}\right\}}_{n}}Q_{{\left\{f_{o}\right\}}_{n}},$$

where $Q_{{\left\{f_{o}\right\}}_{s}}\in C^{M\times K}$, $\Lambda_{{\left\{f_{o}\right\}}_{s}}\in R^{K\times K}$ are the matrix of eigenvectors and diagonal matrix of eigenvalues in signal subspace, and likewise, $Q_{{\left\{f_{o}\right\}}_{n}}\in C^{M\times M-K}$, $\Lambda_{{\left\{f_{o}\right\}}_{n}}\in R^{M-K\times M-K}$ are with noise subspace. In case of $R_{\left\{f,f_{o}\right\}}$, it can be derived by performing SVD, which directly follows from Equation (10). Since $A\left(\phi ,\theta ,f\right)$ and $A\left(\phi ,\theta ,f_{o}\right)$ are full rank matrices [36], its remaining components are expressed as follows [48]:

(A2) $$\begin{array}{cc} U_{{\left\{f,f_{o}\right\}}_{s}}=A\left(\phi ,\theta ,f\right)F_{\left\{f,f_{o}\right\}}^{-1},\;V_{{\left\{f,f_{o}\right\}}_{s}} & =A\left(\phi ,\theta ,f_{o}\right)G_{\left\{f,f_{o}\right\}}^{-1},\;Q_{{\left\{f_{o}\right\}}_{s}}=A\left(\phi ,\theta ,f_{o}\right)H_{\left\{f_{o}\right\}}^{-1}, \end{array}$$

where

(A3) $$\begin{array}{cc} \Sigma_{{\left\{f,f_{o}\right\}}_{s}}=F_{\left\{f,f_{o}\right\}}S_{\left\{f,f_{o}\right\}}G_{\left\{f,f_{o}\right\}}^{H},\; & \;\Lambda_{{\left\{f_{o}\right\}}_{s}}=H_{\left\{f_{o}\right\}}S_{\left\{f_{o},f_{o}\right\}}H_{\left\{f_{o}\right\}}^{H}, \end{array}$$

$F_{\left\{f,f_{o}\right\}},G_{\left\{f,f_{o}\right\}},H_{\left\{f_{o}\right\}}\in C^{K\times K}$ are also full rank and invertible. Note again that $U_{{\left\{f,f_{o}\right\}}_{s}},V_{{\left\{f,f_{o}\right\}}_{s}},Q_{{\left\{f_{o}\right\}}_{s}}$ have orthonormal columns [46], hence, it is obvious to see that

(A4) $$Q_{{\left\{f_{o}\right\}}_{s}}=V_{{\left\{f,f_{o}\right\}}_{s}}G_{\left\{f,f_{o}\right\}}H_{\left\{f_{o}\right\}}^{-1},$$

(A5) $$\begin{array}{cc} H_{\left\{f_{o}\right\}}^{H}H_{\left\{f_{o}\right\}} & =A^{H}\left(\phi ,\theta ,f_{o}\right)A\left(\phi ,\theta ,f_{o}\right)\\ & =G_{\left\{f,f_{o}\right\}}^{H}G_{\left\{f,f_{o}\right\}}. \end{array}$$

From Equation (A4), we may expect that $G_{\left\{f,f_{o}\right\}},H_{\left\{f_{o}\right\}}$ have unitary property, but it is incorrect when considering Equation (A5). Therefore, a proposition of $G_{\left\{f,f_{o}\right\}}H_{\left\{f,f_{o}\right\}}^{-1}$ have to be identity;

(A6) $$\begin{array}{cc} G_{\left\{f,f_{o}\right\}}H_{\left\{f_{o}\right\}}^{-1} & =V_{{\left\{f,f_{o}\right\}}_{s}}^{H}Q_{{\left\{f_{o}\right\}}_{s}}\\ & =I_{K}. \end{array}$$

When considering only the signal subspace, it can be seen from Equations (A4)–(A6) that the right singular vectors of $R_{\left\{f,f_{o}\right\}}$ and the eigenvectors of $R_{\left\{f_{o},f_{o}\right\}}$ are identical.

Next, we continue to generalize the objective function in Equation (15) by utilizing *Orthogonal Procrustes* (OP) [54], but with some modification (MOP). The objective function in Equation (15) is rederived by

(A7) $$\begin{array}{c} {∥R_{\left\{f_{o},f_{o}\right\}}-T_{\left\{f\right\}}R_{\left\{f,f_{o}\right\}}∥}_{F}^{2}\\ =\mathrm{tr}\left(R_{\left\{f_{o},f_{o}\right\}}R_{\left\{f_{o},f_{o}\right\}}^{H}\right)+\mathrm{tr}\left(T_{\left\{f\right\}}R_{\left\{f,f_{o}\right\}}R_{\left\{f,f_{o}\right\}}^{H}T_{\left\{f\right\}}^{H}\right)-2\;\cdot \;ℜ\left(\mathrm{tr}\left(T_{\left\{f\right\}}R_{\left\{f,f_{o}\right\}}R_{\left\{f_{o},f_{o}\right\}}^{H}\right)\right), \end{array}$$

where $ℜ\left(a\right)$ returns the real part of the variable *a*, $\mathrm{tr}\left(A\right)$ trace of the square matrix A. Considering each expression in Equation (A7), the trace of a product of two square matrices is independent of the orders;

(A8) $$\begin{array}{cc} \mathrm{tr}\left(R_{\left\{f_{o},f_{o}\right\}}R_{\left\{f_{o},f_{o}\right\}}^{H}\right) & =\mathrm{tr}\left(\Lambda_{{\left\{f_{o}\right\}}_{s}}\Lambda_{{\left\{f_{o}\right\}}_{s}}\right)+\mathrm{tr}\left(\Lambda_{{\left\{f_{o}\right\}}_{n}}\Lambda_{{\left\{f_{o}\right\}}_{n}}\right). \end{array}$$

Employing Lemma 1, next, we have

(A9) $$\begin{array}{c} \mathrm{tr}\left(T_{\left\{f\right\}}R_{\left\{f,f_{o}\right\}}R_{\left\{f,f_{o}\right\}}^{H}T_{\left\{f\right\}}^{H}\right)\\ =\mathrm{tr}\left(\Sigma_{{\left\{f,f_{o}\right\}}_{s}}\Sigma_{{\left\{f,f_{o}\right\}}_{s}}\right)+\mathrm{tr}\left(\Sigma_{{\left\{f,f_{o}\right\}}_{n}}\Sigma_{{\left\{f,f_{o}\right\}}_{n}}U_{{\left\{f,f_{o}\right\}}_{n}}^{H}T_{\left\{f\right\}}^{H}T_{\left\{f\right\}}U_{{\left\{f,f_{o}\right\}}_{n}}\right), \end{array}$$

From Equation (A6), we finally have

(A10) $$\begin{array}{c} \mathrm{tr}\left(T_{\left\{f\right\}}R_{\left\{f,f_{o}\right\}}R_{\left\{f_{o},f_{o}\right\}}^{H}\right)\\ =\mathrm{tr}\left(\Sigma_{{\left\{f,f_{o}\right\}}_{s}}\Lambda_{{\left\{f_{o}\right\}}_{s}}Q_{{\left\{f_{o}\right\}}_{s}}^{H}T_{\left\{f\right\}}U_{{\left\{f,f_{o}\right\}}_{s}}\right)+\mathrm{tr}\left(\Sigma_{{\left\{f,f_{o}\right\}}_{n}}\Lambda_{{\left\{f_{o}\right\}}_{n}}Q_{{\left\{f_{o}\right\}}_{n}}^{H}T_{\left\{f\right\}}U_{{\left\{f,f_{o}\right\}}_{n}}\right) \end{array}$$

Substituting Equations (A8)–(A10) into Equation (A7), the objective function is simplified as

(A11) $$\begin{array}{cc} {∥R_{\left\{f_{o},f_{o}\right\}}-T_{\left\{f\right\}}R_{\left\{f,f_{o}\right\}}∥}_{F}^{2} & =\mathrm{tr}\left(\Lambda_{{\left\{f_{o}\right\}}_{s}}\Lambda_{{\left\{f_{o}\right\}}_{s}}\right)+\mathrm{tr}\left(\Lambda_{{\left\{f_{o}\right\}}_{n}}\Lambda_{{\left\{f_{o}\right\}}_{n}}\right)+\mathrm{tr}\left(\Sigma_{{\left\{f,f_{o}\right\}}_{s}}\Sigma_{{\left\{f,f_{o}\right\}}_{s}}\right)\\ & \;+\mathrm{tr}\left(\Sigma_{{\left\{f,f_{o}\right\}}_{n}}\Sigma_{{\left\{f,f_{o}\right\}}_{n}}U_{{\left\{f,f_{o}\right\}}_{n}}^{H}T_{\left\{f\right\}}^{H}T_{\left\{f\right\}}U_{{\left\{f,f_{o}\right\}}_{n}}\right)\\ & \;-2\;\cdot \;ℜ\left(\mathrm{tr}\left(\Sigma_{{\left\{f,f_{o}\right\}}_{s}}\Lambda_{{\left\{f_{o}\right\}}_{s}}Q_{{\left\{f_{o}\right\}}_{s}}^{H}T_{\left\{f\right\}}U_{{\left\{f,f_{o}\right\}}_{s}}\right)\right)\\ & \;-2\;\cdot \;ℜ\left(\mathrm{tr}\left(\Sigma_{{\left\{f,f_{o}\right\}}_{n}}\Lambda_{{\left\{f_{o}\right\}}_{n}}Q_{{\left\{f_{o}\right\}}_{n}}^{H}T_{\left\{f\right\}}U_{{\left\{f,f_{o}\right\}}_{n}}\right)\right). \end{array}$$

Three expressions of $\mathrm{tr}\left(\Lambda_{{\left\{f_{o}\right\}}_{s}}\Lambda_{{\left\{f_{o}\right\}}_{s}}\right)$, $\mathrm{tr}\left(\Lambda_{{\left\{f_{o}\right\}}_{n}}\Lambda_{{\left\{f_{o}\right\}}_{n}}\right)$, $\mathrm{tr}\left(\Sigma_{{\left\{f,f_{o}\right\}}_{s}}\Sigma_{{\left\{f,f_{o}\right\}}_{s}}\right)$ are completely isolated from $T_{\left\{f\right\}}$. Therefore, the optimization problem is redefined as

(A12) $$\begin{array}{cc} \underset{T_{\left\{f\right\}}}{\mathrm{minimize}} & \begin{array}{c} \mathrm{tr}\left(\Sigma_{{\left\{f,f_{o}\right\}}_{n}}\Sigma_{{\left\{f,f_{o}\right\}}_{n}}U_{{\left\{f,f_{o}\right\}}_{n}}^{H}T_{\left\{f\right\}}^{H}T_{\left\{f\right\}}U_{{\left\{f,f_{o}\right\}}_{n}}\right)\\ -2\;\cdot \;ℜ\left(\mathrm{tr}\left(\Sigma_{{\left\{f,f_{o}\right\}}_{s}}\Lambda_{{\left\{f_{o}\right\}}_{s}}Q_{{\left\{f_{o}\right\}}_{s}}^{H}T_{\left\{f\right\}}U_{{\left\{f,f_{o}\right\}}_{s}}\right)\right)\\ -2\;\cdot \;ℜ\left(\mathrm{tr}\left(\Sigma_{{\left\{f,f_{o}\right\}}_{n}}\Lambda_{{\left\{f_{o}\right\}}_{n}}Q_{{\left\{f_{o}\right\}}_{n}}^{H}T_{\left\{f\right\}}U_{{\left\{f,f_{o}\right\}}_{n}}\right)\right) \end{array}\\ \mathrm{subject}\;\mathrm{to} & \begin{array}{c} \sum_{k=1}^{K}\sigma_{k}^{2}\left(T_{\left\{f\right\}}R_{\left\{f,f_{o}\right\}}\right)=\sum_{k=1}^{K}\sigma_{k}^{2}\left(R_{\left\{f,f_{o}\right\}}\right). \end{array} \end{array}$$

Now there are two possible cases which we need to consider. The first case is when the M−K smallest singular values of $R_{\left\{f,f_{o}\right\}}$ are close to zeros; the other is when some of the M−K smallest singular values of $R_{\left\{f,f_{o}\right\}}$ are morn than zeros.

Case 1: Assume that all the M−K smallest singular values of $R_{\left\{f,f_{o}\right\}}$ are close to zeros, we have

(A13) $$\begin{array}{cc} \underset{T_{\left\{f\right\}}}{\mathrm{maximize}} & 2\;\cdot \;ℜ\left(\mathrm{tr}\left(\Sigma_{{\left\{f,f_{o}\right\}}_{s}}\Lambda_{{\left\{f_{o}\right\}}_{s}}Q_{{\left\{f_{o}\right\}}_{s}}^{H}T_{\left\{f\right\}}U_{{\left\{f,f_{o}\right\}}_{s}}\right)\right)\\ \mathrm{subject}\;\mathrm{to} & \begin{array}{c} \sum_{k=1}^{K}\sigma_{k}^{2}\left(T_{\left\{f\right\}}R_{\left\{f,f_{o}\right\}}\right)=\sum_{k=1}^{K}\sigma_{k}^{2}\left(R_{\left\{f,f_{o}\right\}}\right),\;\sum_{m=K+1}^{M}\sigma_{m}^{2}\left(R_{\left\{f,f_{o}\right\}}\right)=0. \end{array} \end{array}$$

Using the proposition of Equation (A6) and employing Lemma 1, two possible solutions to reach the maximum point of Equation (A13) can be found. The first solution is given by

(A14) $$T_{{\left\{f\right\}}_{\mathrm{MOP}}}=Q_{{\left\{f_{o}\right\}}_{s}}\Omega_{{\left\{f\right\}}_{\mathrm{MOP}}}U_{{\left\{f,f_{o}\right\}}_{s}}^{†},$$

where orthonormal columns has not yet been defined on $U_{{\left\{f,f_{o}\right\}}_{s}}$, and the second solution is given by

(A15) $$T_{{\left\{f\right\}}_{\mathrm{OP}}}=Q_{{\left\{f_{o}\right\}}_{s}}\Omega_{{\left\{f\right\}}_{\mathrm{OP}}}U_{{\left\{f,f_{o}\right\}}_{s}}^{H},$$

where $U_{{\left\{f,f_{o}\right\}}_{s}}$ has orthonormal columns. Note that the subscript † denotes the pseudo-inverse. When the constraints in Equation (A13) are imposed into Equations (A15) and (A14), we can have that $\Omega_{{\left\{f\right\}}_{\mathrm{MOP}}}=I_{K}$, $\Omega_{{\left\{f\right\}}_{\mathrm{OP}}}=I_{K}$, and the maximum is achieved;

(A16) $${\bar{\varrho }}_{{\left\{f\right\}}_{\mathrm{case}\;1}}=2\;\cdot \;\mathrm{tr}\left(\Sigma_{{\left\{f,f_{o}\right\}}_{s}}\Lambda_{{\left\{f_{o}\right\}}_{s}}\right).$$

Case 2: Assume that some of the M−K smallest singular values of $R_{\left\{f,f_{o}\right\}}$ are more than zeros, the best solution of Equation (A12) can be given the same as Equation (A15), and its minimum is equal to Equation (A16);

(A17) $$\begin{array}{cc} {\underline{\varrho }}_{{\left\{f\right\}}_{\mathrm{case}\;2:\mathrm{OP}}} & =-2\;\cdot \;\mathrm{tr}\left(\Sigma_{{\left\{f,f_{o}\right\}}_{s}}\Lambda_{{\left\{f_{o}\right\}}_{s}}\right). \end{array}$$

On the contrary, when using Equation (A14) in Equation (A12), the minimum of cost function is remained by

(A18) $$\begin{array}{cc} {\underline{\varrho }}_{{\left\{f\right\}}_{\mathrm{case}\;2:\mathrm{MOP}}} & =\mathrm{tr}\left(\Sigma_{{\left\{f,f_{o}\right\}}_{n}}\Sigma_{{\left\{f,f_{o}\right\}}_{n}}{\left(U_{{\left\{f,f_{o}\right\}}_{s}}^{†}U_{{\left\{f,f_{o}\right\}}_{n}}\right)}^{H}U_{{\left\{f,f_{o}\right\}}_{s}}^{†}U_{{\left\{f,f_{o}\right\}}_{n}}\right)-2\;\cdot \;\mathrm{tr}\left(\Sigma_{{\left\{f,f_{o}\right\}}_{s}}\Lambda_{{\left\{f_{o}\right\}}_{s}}\right). \end{array}$$

Using the solution of Equation (A14) rather than Equation (A15) allows us to relax the error constraint in the hope of arriving at a reduction in the computation of HOGSVD (For details, see Section 3.2), but this is still sufficient for estimating $T_{\left\{f\right\}}$ without loss of generality; the squares of M−K smallest singular values of $R_{\left\{f,f_{o}\right\}}$ are very close to zeros, so we can assume that $\Sigma_{{\left\{f,f_{o}\right\}}_{n}}^{2}\approx O_{M-K\times M-K}$. Remark that error of the transformation remains consistent with the following equation;

(A19) $$\begin{array}{cc} \varepsilon_{{\left\{f\right\}}_{\mathrm{MOP}}} & =\left|2\;\cdot \;ℜ\left(\mathrm{tr}\left(\Sigma_{{\left\{f,f_{o}\right\}}_{s}}\Lambda_{{\left\{f_{o}\right\}}_{s}}\left(\Omega_{\left\{f\right\}}-I_{K}\right)\right)\right)\right.\\ & \;\;\left.+\mathrm{tr}\left(\Sigma_{{\left\{f,f_{o}\right\}}_{n}}^{2}{\left(U_{{\left\{f,f_{o}\right\}}_{s}}^{†}U_{{\left\{f,f_{o}\right\}}_{n}}\right)}^{H}U_{{\left\{f,f_{o}\right\}}_{s}}^{†}U_{{\left\{f,f_{o}\right\}}_{n}}\right)\right|. \end{array}$$

To further reduce a computational burden caused by performing SVD of $R_{\left\{f,f_{o}\right\}}$ and EVD of $R_{\left\{f_{o},f_{o}\right\}}$, we reinitialize the cross-correlation matrix as

(A20) $$\begin{array}{c} R_{\left\{f,f_{o}\right\}}R_{\left\{f_{o},f_{o}\right\}}^{H}\\ =\left(U_{{\left\{f,f_{o}\right\}}_{s}}\Sigma_{{\left\{f,f_{o}\right\}}_{s}}V_{{\left\{f,f_{o}\right\}}_{s}}^{H}+U_{{\left\{f,f_{o}\right\}}_{n}}\Sigma_{{\left\{f,f_{o}\right\}}_{n}}V_{{\left\{f,f_{o}\right\}}_{n}}^{H}\right){\left(Q_{{\left\{f_{o}\right\}}_{s}}\Lambda_{{\left\{f_{o}\right\}}_{s}}Q_{{\left\{f_{o}\right\}}_{s}}^{H}+Q_{{\left\{f_{o}\right\}}_{n}}\Lambda_{{\left\{f_{o}\right\}}_{n}}Q_{{\left\{f_{o}\right\}}_{n}}^{H}\right)}^{H}\\ =U_{{\left\{f,f_{o}\right\}}_{s}}\Sigma_{{\left\{f,f_{o}\right\}}_{s}}\Lambda_{{\left\{f_{o}\right\}}_{s}}Q_{{\left\{f_{o}\right\}}_{s}}^{H}+U_{{\left\{f,f_{o}\right\}}_{n}}\Sigma_{{\left\{f,f_{o}\right\}}_{n}}\Lambda_{{\left\{f_{o}\right\}}_{n}}Q_{{\left\{f_{o}\right\}}_{n}}^{H}, \end{array}$$

which is possible to reduce the computation by performing single SVD operation on $R_{\left\{f,f_{o}\right\}}R_{\left\{f_{o},f_{o}\right\}}^{H}$.
